# Historical biogeography of the genus *Rhadinaea* (Squamata: Dipsadinae)

**DOI:** 10.1002/ece3.7988

**Published:** 2021-08-05

**Authors:** Uriel A. García‐Sotelo, Uri O. García‐Vázquez, David Espinosa

**Affiliations:** ^1^ Posgrado en Ciencias Biológicas Universidad Nacional Autónoma de México Ciudad de México Mexico; ^2^ Facultad de Estudios Superiores Zaragoza Universidad Nacional Autónoma de México Ciudad de México Mexico

**Keywords:** ancestral area reconstruction, divergence dating, Mexican Transition Zone, Pleistocenic Climate Change, Sierra Madre del Sur, Trans‐Mexican Volcanic Belt

## Abstract

Multiple geological and climatic events have created geographical or ecological barriers associated with speciation events, playing a role in biological diversification in North and Central America. Here, we evaluate the influence of the Neogene and Quaternary geological events, as well as the climatic changes in the diversification of the colubrid snake genus *Rhadinaea* using molecular dating and ancestral area reconstruction. A multilocus sequence dataset was generated for 37 individuals of *Rhadinaea* from most of the biogeographical provinces where the genus is distributed, representing 19 of the 21 currently recognized species, and two undescribed species. Our analyses show that the majority of the *Rhadinaea* species nest in two main clades, herein identified as “Eastern” and “Southern”. These clades probably diverged from each other in the early Miocene, and their divergence was followed by 11 divergences during the middle to late Miocene, three divergences during the Pliocene, and six divergences in the Pleistocene. The ancestral distribution of *Rhadinaea* was reconstructed across the Sierra Madre del Sur. Our phylogenetic analyses do not support the monophyly of *Rhadinaea*. The Miocene and Pliocene geomorphology, perhaps in conjunction with climate change, appears to have triggered the diversification of the genus, while the climatic changes during the Miocene probably induced the diversification of *Rhadinaea* in the Sierra Madre del Sur. Our analysis suggests that the uplifting of the Trans‐Mexican Volcanic Belt and Chiapan–Guatemalan highlands in this same period resulted in northward and southward colonization events. This was followed by more recent, independent colonization events in the Pliocene and Pleistocene involving the Balsas Basin, Chihuahuan Desert, Pacific Coast, Sierra Madre Occidental, Sierra Madre Oriental, Sierra Madre del Sur, Trans‐Mexican Volcanic Belt, and Veracruz provinces, probably driven by the climatic fluctuations of the time.

## Graphical abstract

Here, we evaluate the influence of Neogene and Quaternary geological events and climatic changes in the diversification of the colubrid snake genus *Rhadinaea* using molecular dating and ancestral area reconstruction.

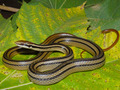

## 1 INTRODUCTION

Inferring the evolutionary history of groups in a particular region is the first step in elucidating the processes by which the region's fauna originated (Colston et al., 2013). Multiple biogeographical and phylogeographical studies of groups with broad distributions through North and Central America are illustrative in this context (e.g., Bryson et al., 2011; Burbrink et al., 2008; Fritz et al., 2012; Hofmann & Townsend, 2017; Phillips et al., 2015). Within this area, the Mexican Transition Zone (MTZ) is a complex, distinguishable area where Neotropical and Nearctic biotas overlap, spanning the region from the southwestern deserts of the United States and northern Mexico to the dry and humid forests of the Nicaraguan lowlands (Morrone, 2010).

Across the North and Central American regions, multiple geological and climatic events resulting from a complex orogeny and paleoclimatic conditions acted as geographical or ecological barriers associated with the speciation and diversification of many taxa (e.g., Bryson, et al., 2011; Daza et al., 2010; Ferrusquía‐Villafranca & González‐Guzmán, 2005; Vanzolini, 1970). Some of the events that are considered of major importance or that have received the most attention are as follows: (a) The Mississippi River Basin (MR) (Burbrink et al., 2000), which was involved in the divergence of many marine and terrestrial taxa during the Pleistocene (Soltis et al., 2006); (b) the last formation of three of the four major mountain ranges in Mexico (i.e., the Sierra Madre Occidental [SMOc], Sierra Madre Oriental [SMOr], and Sierra Madre del Sur [SMS]; Ferrusquía‐Villafranca & González‐Guzmán, 2005) during the Paleogene and early Neogene (Padilla y Sánchez, 2007), which probably predate the origin of most extant species (Bryson et al., 2012); (c) the formation of the Trans‐Mexican Volcanic Belt (TVB) during the Neogene (~20–1 million years [Ma]) (Ferrari et al., 2012; Gómez‐Tuena et al., 2007) in four major orogenic events (Ferrari et al., 2012) that undoubtedly affected both the timing and tempo of the biota diversification (Bryson et al., 2012a, 2012b); (d) the faulting and marine introgressions across the Isthmus of Tehuantepec (IT) in southeastern Mexico around 3 Ma (Mulcahy et al., 2006), a region which is a narrow lowland area that has been identified as a biogeographical barrier for many upland taxa (Castoe et al., 2009); (e) the Nicaraguan depression (ND), an area that presented different states of terrestrial conformation during the Neogene (2.5–23 Ma) (Funk et al., 2009) and probably presented a lowland biogeographical barrier to some taxa (Daza et al., 2010); (f) the Panama Isthmus in southern Central America, another narrow area that was completely conformed during the Pliocene (3.5 Ma), which has separated numerous taxa between Central and South America (Mendoza et al., 2019); and (g) the climatic fluctuations during the Pleistocene (0.01–2.5 Ma) (Vanzolini, 1970) that conditioned the diversification of a variety of taxa across the American continent through the repeated expansion and contraction of coniferous forests, leading isolated populations of forest‐adapted taxa to speciation (Haffer, 1969, 1997).

These events, in addition to other physiographic conditions, such as river drainages within the major sierras, basins, and faults, are considered to act as biogeographical barriers (Bryson, Murphy, et al., 2011; Bryson et al., 2007; Daza et al., 2010; León‐Paniagua et al., 2007), yet the effectiveness of these barriers in isolating lineages throughout the past several million years remains to be clarified (Bryson et al., 2012a, 2012b; García‐Vázquez et al., 2018).

**FIGURE 1:**
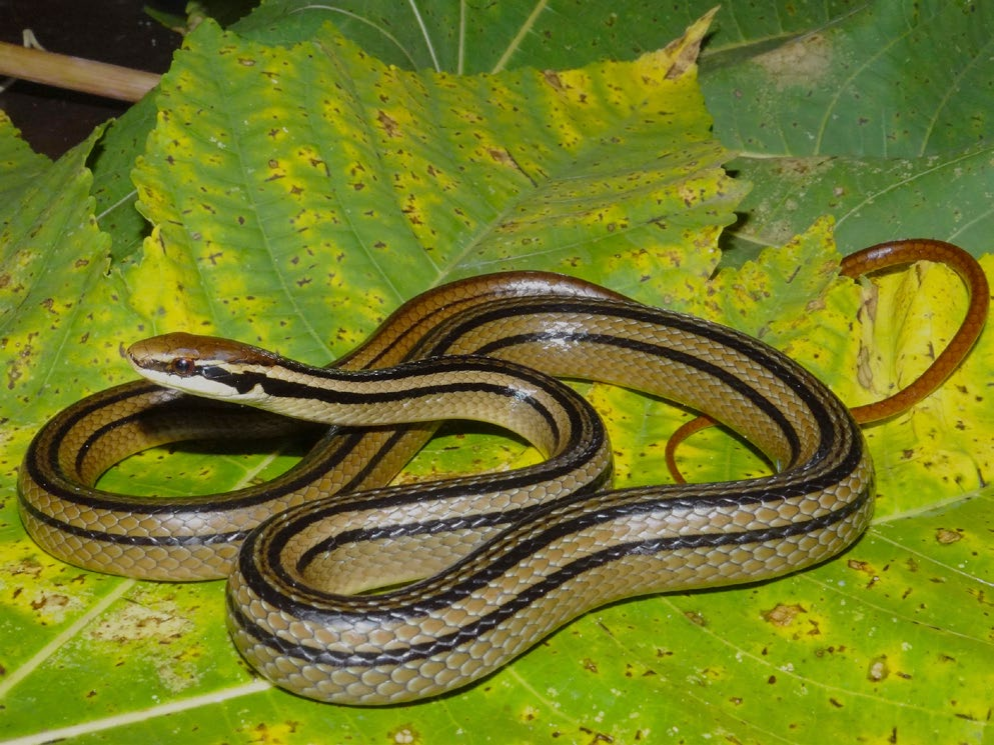
A living specimen of *Rhadinaea taeniata*, spotted in Sierra del Tigre, Quitupan, Jalisco, in 2013. Photography by Christoph I. Grünwald

The colubrid snakes of the genus *Rhadinaea* are slender, diurnal, medium‐ to small‐sized snakes that are characterized by longitudinal dark stripes along the dorsal scales (Figure 1), a small subpreocular scale inserted between the corners of two supralabial scales at the anteroventral edge of the orbit, the same number of longitudinal dorsal scale rows throughout the body, and maxillary teeth without grooves posterior to the diastema (Myers, 1974, 2011; Palacios‐Aguilar & García‐Vázquez, 2020). Currently, 21 species of *Rhadinaea* are recognized and arranged into five morphological groups (García‐Vázquez et al., 2018; Myers, 2011; Palacios‐Aguilar & García‐Vázquez, 2020). The genus is present in North and Central America from southeastern United States to Panama, with discontinuities across the Chihuahuan Desert, southern Guatemala, El Salvador, Honduras, and central Nicaragua (Table 1) (Myers, 1974, 2011). The most speciose and widely distributed of these groups is the decorata group, represented by 12 species mainly distributed over the Mexican Sierras (García‐Vázquez, 2012; García‐Vázquez et al., 2009; García‐Vázquez, Pavón‐Vázquez, et al., 2018; Luría‐Manzano et al., 2014; Pérez‐Higareda et al., 2002; Sánchez‐García et al., 2019; Torres‐Carvajal et al., 2019); the taeniata group is endemic to Mexico and is composed of three species distributed in central Mexico (Canseco‐Márquez & Gutiérrez‐Mayén, 2010; García‐Sotelo et al., 2018; García‐Vázquez, Pavón‐Vázquez, et al., 2018; Myers, 1974); the flavilata group is comprised of two species with allopatric distributions in North America (Auth et al., 1999; Lares et al., 2013; Walley, 1999); and the calligaster and vermiculaticeps groups are restricted to Central America (Myers, 1974). In addition to these 21 species, the existence of two undescribed species has been suggested based on their morphology and previous analyses (pers. obs.).

**TABLE 1:** Distribution of the species of *Rhadinaea* corresponding with the biogeographic regionalization of Mexico (Morrone et al., 2017), Neotropical America (Löwenberg‐Neto, 2014), and North America (Escalante et al., 2013)

Group	Species	Morrone et al. (2017)									Löwenberg‐Neto (2014)					Escalante et al. (2013)
		SMOr	VER	CHIH	TVB	PAC	BB	SMS	CHIS	SMOc	CHIS‐H	PAC‐C	G‐T	P‐CH	CH‐DA	ALLE
Decorata	*R. bogertorum*							X								
	*R. cuneata*		X					X								
	*R. decorata*	X	X			X		X	X		X	X	X	X	X	
	*R. forbesi*				X			X								
	*R. gaigeae*	X														
	*R. hesperia*			X	X	X	X	X		X						
	*R. macdougalli*		X					X								
	*R. marcellae*	X	X													
	*R. montana*	X	X													
	*R. myersi*							X								
	*R. nuchalis*							X								
	*R. quinquelineata*	X			X											
Taeniata	*R. fulvivittis*							X								
	*R. omiltemana*							X								
	*R. taeniata*				X		X	X								
Flavilata	*R. flavilata*															X
	*R. laureata*				X			X		X						
Vermiculaticeps	*R. pulveriventris*												X	X		
	*R. sargenti*												X			
	*R. vermiculaticeps*												X	X		
Calligaster	*R. calligaster*												X	X		

Note

Alleghanian Region (ALLE); Balsas Basin (BB); Chocó‐Darién (CH‐DA); Chiapas (CHIS); Chiapas Highlands (CHIS‐H); Chihuahuan Plateau (CHIH); Gatuso‐Talamanca (G‐T); Pacific Lowlands (PAC); Pacific coast (PAC‐C); Puntarenas–Chiriquí (P‐CH); Sierra Madre Occidental (SMOc); Sierra Madre Oriental (SMOr); Sierra Madre del Sur (SMS); Trans‐Mexican Volcanic Belt (TVB); and Veracruz (VER). The CHIS and CHIS‐H provinces, as well as PAC and PAC‐C, represent homologous biogeographic regions that overlap in both regionalizations.

The systematics of *Rhadinaea* has been poorly studied, and, to date, the arrangements of Myers (2011) are predominant. A recent study (Palacios‐Aguilar & García‐Vázquez, 2020) using a mitochondrial fragment of DNA provides a comprehensive insight into the phylogenetic relationships of this Neotropical snake genera, supporting the reciprocal monophyly of *Rhadinaea* and *Rhadinella* (a former *Rhadinaea* group separated by morphological evidence by Myers [2011]), and a close relationship with *Coniophanes* as a sister group (as previously suggested by several authors; e.g., Bailey, 1939; Cadle, 1984; Myers, 1974; Zaher et al., 2019). Additionally, *Rhadinophanes* is a monotypic genus that is considered the sister group of *Rhadinaea* and *Coniophanes* (Cadle, 1984; Myers & Campbell, 1981), a relationship that has not been tested using molecular data. To explore their monophyly and evolutionary history, Palacios‐Aguilar and García‐Vázquez (2020) suggest that it is necessary to include more representatives of the genera using more molecular markers.

To date, the five groups composing *Rhadinaea* are sorted out based on their color patterns, which are the most informative character to distinguish them, even among species within *Rhadinaea* (Myers, 1974, 2011). However, the reciprocal monophyly of the groups is still to be assessed (García‐Vázquez, Pavón‐Vázquez, et al., 2018). On the other hand, according to Myers (1974), the origin of *Rhadinaea* is related to the dispersal of an ancestor related to *Rhadinella* (former godmani group), in which geographical isolation and the subsequent evolution of terminal populations occurred, probably after unfavorable climatic changes or flooding that created barriers of lowland regions, such as the IT and the ND (Myers, 1974); yet, other geographical barriers corresponding to the distribution of the genus, such as the formation of the TVB or climatic events, are not discussed.

In this work, we describe the phylogenetic relationships of *Rhadinaea* to evaluate the role of major orogenic events and Pleistocene climatic fluctuations on lineage diversification. Samples of the five recognized groups and two undescribed species of *Rhadinaea* were included (*R*. cf. *marcellae* and *R*. cf. *taeniata*). Two mitochondrial and two nuclear loci were sequenced. We inferred the phylogenetic relationships and a time‐calibrated tree from these data. Finally, ancestral ranges were reconstructed at each divergence event. The resulting patterns of diversification are discussed in the context of mountain formations and climatic change.

## 2 MATERIALS AND METHODS

### 2.1 Taxon sampling and laboratory methods

**FIGURE 2:**
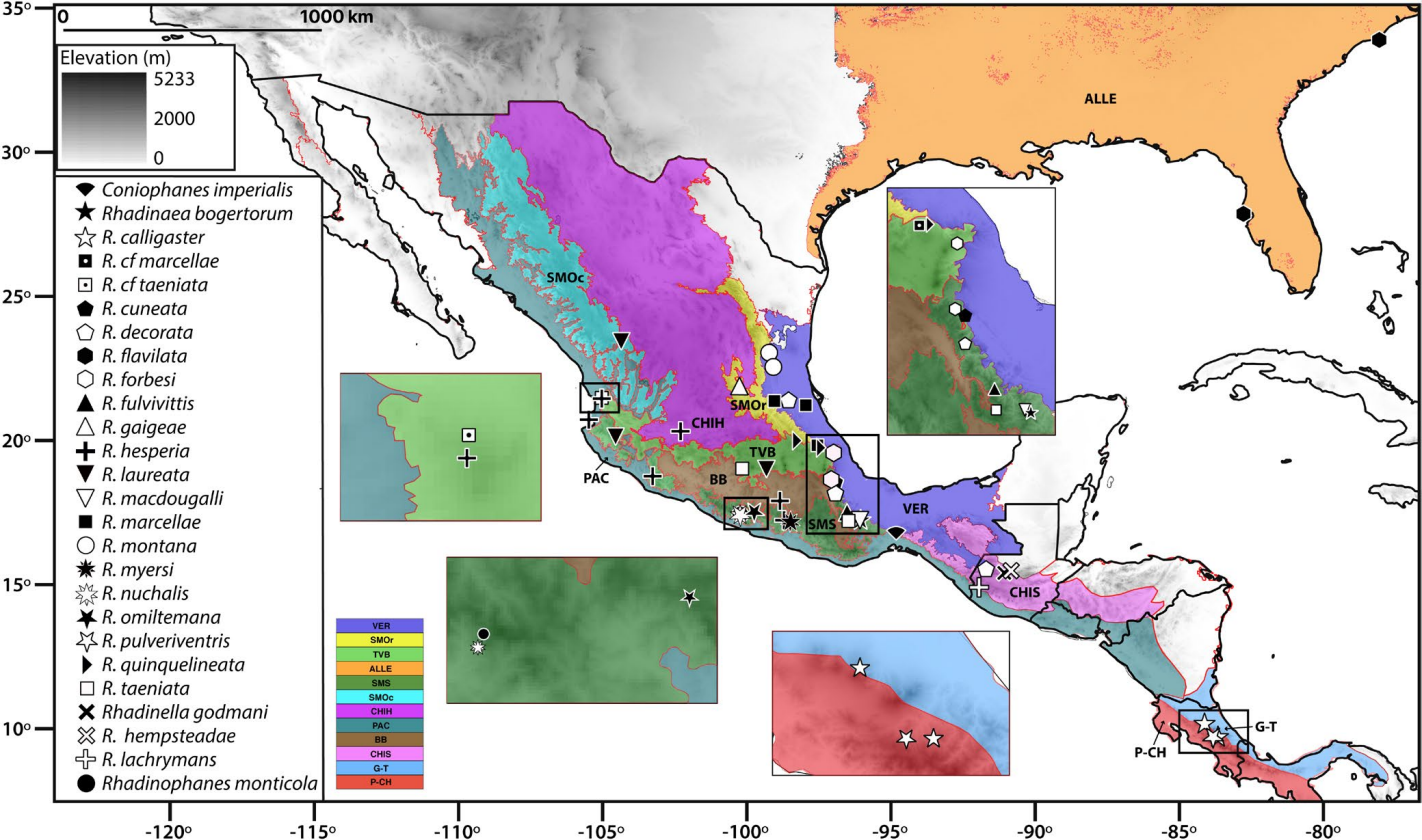
Sampling localities for the genetic samples used in this study (see Appendix S1). Black lines indicate political boundaries; red lines indicate biogeographic regions (Escalante et al., 2013; Löwenberg‐Neto, 2014; Morrone et al., 2017). Alleghanian Region (ALLE); Balsas Basin (BB); Chiapas (CHIS); Chihuahuan Plateau (CHIH); Gatuso‐Talamanca (G‐T); Pacific Lowlands (PAC); Puntarenas‐Chiriquí (P‐CH); Sierra Madre Occidental (SMOc); Sierra Madre Oriental (SMOr); Sierra Madre del Sur (SMS); Trans‐Mexican Volcanic Belt (TVB); and Veracruz (VER)

In this study, we cover 37 samples of *Rhadinaea*, including most of the currently recognized species of the genus (Figure 2; Appendix S1), with the exception of *R. sargenti* and *R. vermiculaticeps*, two species that are rarely found within biological collections. Samples from two undescribed species from the TVB were also included. To increase geographic representativeness for species with a wide distribution range, a sample per biotic region was included, following the biogeographical regionalization of North America (Escalante et al., 2013), Mexico (Morrone et al., 2017), and Neotropical America (Löwenberg‐Neto, 2014).

In order to test the monophyly of *Rhadinaea*, we added one representative of *Coniophanes imperialis, Rhadinella godmani, Rhadinella hempsteade*, *Rhadinella lachrymans,* and *Rhadinophanes monticola* given the evidence that they are close relatives to this genus (Cadle, 1984; Myers, 1974, 2011; Myers & Campbell, 1981). Finally, to time‐calibrate our phylogenetic tree, sequences from three colubrid genera and two close relatives within Caenophidia were incorporated (Grazziotin et al., 2012; Pyron et al., 2011; Zaher et al., 2019; Appendix S1).

Partial sequences of the mitochondrial gene coding for the cytochrome b (*cytb*), NADH dehydrogenase subunit 4 (*ND4*); complete sequences of the noncoding tRNA‐His and tRNA‐Ser; partial sequences of the noncoding tRNA‐Leu; partial sequences of the nuclear genes coding for oocyte maturation factor (*cmos*) and dynein axonemal heavy chain 3 (*DNAH3*) were obtained for all the 37 individuals of *Rhadinaea* and five outgroup individuals. Loci were selected as they previously showed to be informative at different levels of divergence between snakes (Bryson, García‐Vázquez, et al., 2011; Bryson, Murphy, et al., 2011; Lawson et al., 2005; Myers et al., 2017). Primer sequences for *cmos* were given by Lawson et al. (2005) and Saint et al. (1998); for *cytb* by Burbrink et al. (2000), de Queiroz et al. (2002), and Slowinski and Lawson (2002); for *DNAH3* by Townsend et al. (2008); and for *ND4* by Arévalo et al. (1994), Forstner et al. (1995). Also, additional internal primers were designed in this study. See Appendix S2 for primer sequences, technical details on DNA sequencing, and sequence edition.

### 2.2 Phylogenetic inference

With regard to testing incongruences among mitochondrial and nuclear loci, we performed Bayesian inference (BI) and maximum‐likelihood (ML) separate analyses for each nuclear and mitochondrial locus. The best‐fitting substitution models and partition schemes were selected jointly using the Bayesian information criterion in the software PartitionFinder 2 (Lanfear et al., 2016). BI analysis was conducted using MrBayes 3.2.7a (Ronquist et al., 2012) with four Monte Carlo Markov chains (MCMC), sampling every 5,000 generations for 100 million generations. Output parameters were visualized using Tracer 1.7.1 (Rambaut et al., 2018) to ascertain stationarity and convergence. The first 25% of generations were discarded as burn‐in to obtain a majority rule consensus tree using the command sumt. ML analysis was conducted using raxmlGUI (Silvestro & Michalak, 2012) under the GTRGAMMA model (Stamatakis, 2006) with 1,000 nonparametric bootstrap replicates to assess nodal support.

Additionally, all nuclear and mitochondrial datasets were combined into one dataset after independent analyses established the congruence between topologies and levels of support between the IB and the ML analyses, using the same partitions and models of evolution suggested by PartitionFinder 2 (Lanfear et al., 2016), and the concatenated matrix was analyzed under BI and ML approaches following the above specifications and settings in each program. Nodes were considered strongly supported if their Bayesian posterior probability (pp) was ≥0.95 and their bootstrap (bs) value was ≥70% (Huelsenbeck & Rannala, 2004).

### 2.3 Divergence times

Divergence dates and phylogeny were estimated simultaneously using a relaxed Bayesian molecular clock framework implemented in BEAST 2.4.8 (Bouckaert et al., 2014) using the concatenated dataset. For this purpose, our multilocus dataset was analyzed with an uncorrelated lognormal clock and node constraints obtained from the fossil record under lognormal distributions, also including a single representative of each species, except for *R. gaigeae*, *R. montana,* and *R. quinquelineata* (see Discussion), based on the completeness of the samples and variation within the sampled sequences (<9.2% uncorrected *p* distance values). The partitions and models for this analysis were estimated using bModelTest (Bouckaert & Drummond, 2017). Four fossil calibration points of Colubridae and related groups were used (see Appendix S3 for technical details on calibration points), while analyses were run for 100 million generations; samples were retained every 5,000 generations and a Yule birth‐death prior was specified. Results were displayed in Tracer 1.7.1 to confirm the proper mixing and likelihood stationarity of the MCMC analyses, appropriate burn‐in, as well as adequate, effective sample sizes (>200 for each estimated parameter). After discarding the initial 20% of the samples as burn‐in, the parameter values of the posterior samples were summarized on a maximum clade credibility tree using TreeAnnotator 1.8.2 (Rambaut & Drummond, 2014), setting the posterior probability limit to 0.1, and summarizing mean node heights.

### 2.4 Ancestral area reconstruction

Ancestral ranges at each divergence event were reconstructed using the Bayesian binary Monte Carlo analysis (BBM) and dispersal–extinction–cladogenesis (DEC) implemented in RASP 2.0 (Yu et al., 2011). This program can determine the probability of an ancestral range at a node by averaging a posterior set of trees and thereby accounting phylogenetic uncertainty (Bryson et al., 2013). A total of 16,000 post‐burn‐in trees and the condensed maximum clade credibility tree obtained in the BEAST analysis were loaded from the divergence time analysis into RASP, removing the outgroups and the *Rhadinella* clade using the “remove selected groups” tool that is implemented in the same program. According to the distribution of each species (Table 1), each sample of the calibrated phylogeny was assigned to the following 14 terminal biogeographical regions (Escalante et al., 2013; Löwenberg‐Neto, 2014; Morrone, 2017): (1) Alleghanian Region (ALLE); (2) Balsas Basin (BB); (3) Chiapas (CHIS); (4) Chihuahuan Plateau (CHIH); (5) Chocó‐Darién (CH‐DA); (6) Gatuso‐Talamanca (G‐T); (7) Pacific Lowlands (PAC); (8) Puntarenas‐Chiriquí (P‐CH); (9) Sierra Madre Occidental (SMOc); (10) Sierra Madre Oriental (SMOr); (11) Sierra Madre del Sur (SMS); (12) Trans‐Mexican Volcanic Belt (TVB); (13) Veracruz (VER); and (14) Yucatán (YUC). The probabilities for the nodes in the phylogeny were estimated, whereas the analysis was conducted for 10 million generations by sampling each 1,000 using ten chains, and the first 25% of the generations were discarded as burn‐in for BBM. For DEC analysis, we set the dispersal rate all equal among the defined areas, performing 100 replicas.

## 3 RESULTS

### 3.1 Phylogenetic inference

Individual genealogies obtained using IB and ML did not share the same topology, as these genealogies show numerous nonsupported nodes and polytomies; however, we did not observe discordances between ML and IB reconstructions for each gene in shared supported nodes, where all of these were congruent (Appendix S4). Due to this situation, we assumed that the phylogenetic information that each sequence dataset provided could be combined to obtain a more accurate phylogeny, whereas congruent data can be combined to yield phylogenies that do not represent organismal history accurately (Cunningham, 1997; Hipp et al., 2004).

**FIGURE 3:**
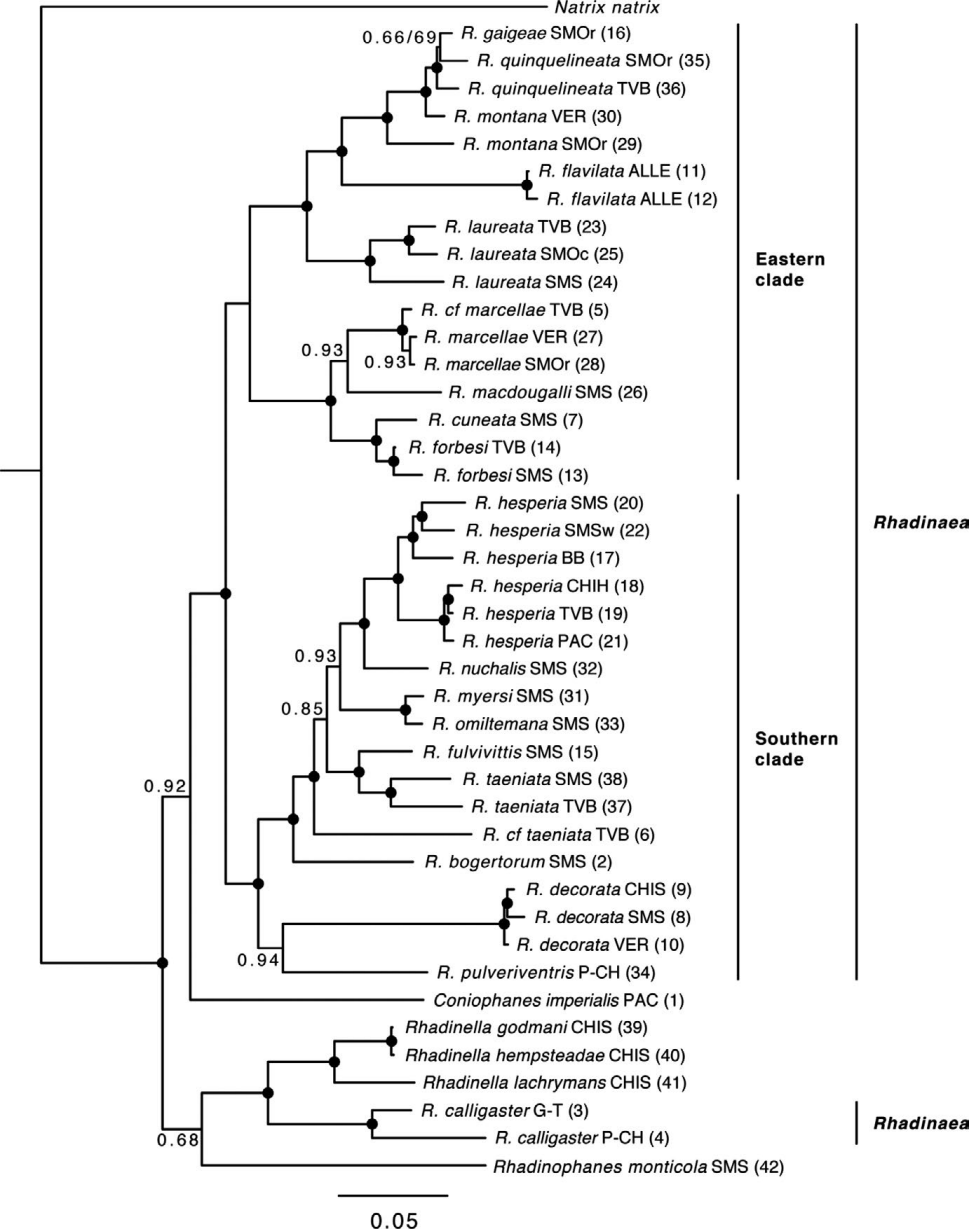
Phylogeny of *Rhadinaea* (BI analyses are shown) and close groups inferred from maximum‐likelihood and Bayesian inference analyses from the DNA concatenated matrix. Black dots represent strongly supported nodes (bootstrap value ≥70; Bayesian posterior probability value ≥0.95). Numbers at other nodes are bootstrap/Bayesian posterior probability values for poorly supported nodes in one or both analyses (marked with a slash)

The final concatenated dataset consisted of 2,899 aligned nucleotide positions. The partitions and models that best fitted the data were as follows: *cytb* third position and *DNAH3* third position, GTR+G; *cytb* second position, tRNA‐His, *ND4* first position and *ND4* third position, GTR+I+G; *cytb* second position and *ND4* second position, TVM+I+G; tRNA‐Ser, TVMEF+G; tRNA‐Leu, TVMEF+G; *cmos* first position and *DNAH3* first position, TRN+I+G; *cmos* second position, TRN+I; *cmos* third position, HKY+I; and *DNAH3* second position, K81UF+I. All sequences were deposited in GenBank (Appendix S1). ML and BI concatenated analyses resulted in highly congruent phylogenetic trees, presenting the same topology strongly supporting most clades (Figure 3).

Our phylogenetic analyses did not support the monophyly of *Rhadinaea* (Figure 3), showing *Rhadinaea calligaster* as the sister taxon of *Rhadinella*, a relationship strongly supported in both analyses. The rest of the genus species were grouped into two strongly supported main clades, which are defined below: an eastern clade (Eastern clade, Figure 3) composed of mostly eastern species (*Rhadinaea cuneata, R*. *flavilata, R. forbesi, R. gaigeae, R. laureata, R. macdougalli, R*. cf. *marcellae, R. marcellae, R. montana,* and *R. quinquelineata*), distributed across the ALLE, SMOc, SMOr, SMS, TVB, and VER; and a southern clade (southern clade, Figure 3) composed mostly of western and southern species (*Rhadinaea bogertorum, R. decorata, R. fulvivittis, R. hesperia, R. myersi, R. nuchalis, R. omiltemana, R. pulveriventris, R. taeniata,* and *R*. cf. *taeniata*), distributed across the BB, CH‐DA, CHIH, CHIS, G‐T, P‐CH, PAC, SMOr, SMOc, SMS, TVB, and VER.

Within the Eastern clade, *R. quinquelineata* is paraphyletic with respect to *R. gaigeae,* and *R. montana* is paraphyletic with respect to *R. gaigeae* and *R. quinquelineata*, with a low support value in both analyses (Figure 3). These species represent the sister group of *R. flavilata*, and in turn, these last haplotypes represent the sister group of *R. laureata*; the sampled haplotypes of *R. marcellae* are recovered as monophyletic with a low support value in the BI analysis (Figure 3) and represent the sister group of *R*. cf. *marcellae*. These species are recovered as the sister group of *R. macdougalli* with a low support value in the BI analysis (Figure 3); and these species represent the sister group of *R. cuneata* + *R*. *forbesi*. Finally, the clade composed of *R. flavilata, R. gaigeae, R. laureata, R. montana,* and *R. quinquelineata* represents the sister group of the clade composed by *R. cuneata, R. forbesi, R. macdougalli, R. marcellae,* and *R*. cf. *marcellae*, a relationship that is strongly supported (Figure 3).

Within the southern clade, *R. hesperia* + *R*. *nuchalis* represent the sister group of *R. myersi* + *R*. *omiltemana*, with a low support value in the BI analysis (Figure 3). In turn, all of these species represent the sister group of *R. fulvivittis* + *R*. *taeniata* with a low support value in the BI analysis (Figure 3). *R*. cf. *taeniata* represents the sister group of these mentioned species as well as *R. bogertorum*. On the other hand, *R. decorata* is recovered as the sister group of *R. pulveriventris* with a low support value in the BI analysis (Figure 3). Finally, the clade composed by *R. bogertorum, R. fulvivittis, R. hesperia, R. myersi, R. nuchalis, R. omiltemana, R. taeniata,* and *R*. cf. *taeniata* represents the sister group of *R. decorata* + *R*. *pulveriventris*, a relationship that is strongly supported (Figure 3).

*Coniophanes* is observed as the sister group of *Rhadinaea* (except *R. calligaster*), which is a relationship strongly supported only in ML analysis. *Rhadinophanes monticola* is recovered as a sister group of *Rhadinaea calligaster* + *Rhadinella*, and this relationship is only supported in the ML analysis (Figure 3).

### 3.2 Divergence times

**FIGURE 4:**
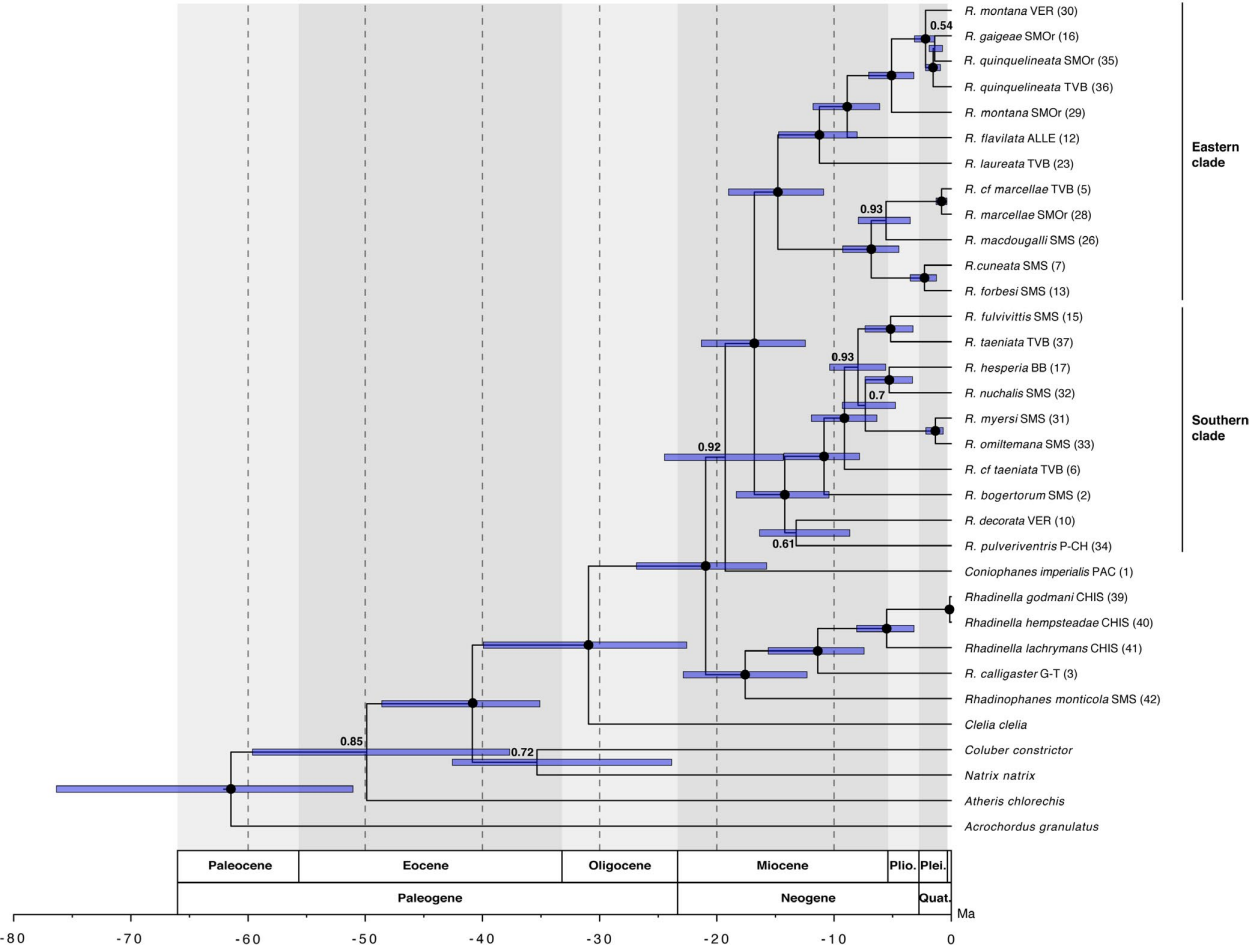
Divergence time estimates for *Rhadinaea* from the multilocus dataset in BEAST. Bars indicate 95% highest posterior densities of divergence dates, with mean estimates in a million years ago (Ma) at the nodes. Nodes with black dots are strongly supported nodes, and numbers near other nodes are Bayesian posterior probability values for poorly supported nodes. Alleghanian Region (ALLE); Balsas Basin (BB); Chiapas (CHIS); Gatuso‐Talamanca (G‐T); Pacific Lowlands (PAC); Puntarenas‐Chiriquí (P‐CH); Sierra Madre Oriental (SMOr); Sierra Madre del Sur (SMS); Trans‐Mexican Volcanic Belt (TVB); and Veracruz (VER)

Our multilocus analysis produced a reconstruction for *Rhadinaea* with moderate resolution and node support (75% of nodes with pp > 0.95). In the calibrated tree, the same clades as phylogenetic analyses were recovered, as well as the relationships between the clades. The dated phylogeny suggests that the diversification of *Rhadinaea* (except *Rhadinaea calligaster*) probably began in the early Miocene (20.9 Ma) (Figure 4) with a basal divergence between major clades (Eastern clade and southern clade) in the early Miocene (16.8 Ma). Several divergences appear to have occurred during the Miocene within Eastern and southern clades (Figure 4). In the Eastern clade, a basal divergence is observed between the clade composed of *R. cuneata, R. forbesi, R. macdougalli, R. marcellae,* and *R*. cf. *marcellae*, and the clade consisting of *R. flavilata, R. gaigeae, R. laureata, R. montana,* and *R. quinquelineata* (14.3 Ma), followed by four splits among these species. In the southern clade, a basal divergence is observed between the clade composed of *R. bogertorum, R. fulvivittis, R. hesperia, R. myersi, R. nuchalis, R. omiltemana, R. taeniata,* and *R*. cf. *taeniata*, and the clade consisting of *R. decorata* and *R. pulveriventris* (14.2 Ma), followed by five splits among these species. Our estimates placed the remaining divergences within the Eastern and southern clades during the Pliocene and Pleistocene (Figure 4).

The divergence time estimates also show the divergence between *Rhadinaea* +Coniophanes and *Rhadinaea calligaster* + *Rhadinella* + *Rhadinophanes* during the early Miocene (21 Ma) (Figure 4), and the divergence between *Rhadinaea calligaster* and *Rhadinella* occurs during the middle Miocene (11.4 Ma) (Figure 4).

### 3.3 Ancestral area reconstruction

The BBM and DEC analyses using the dated multilocus phylogeny obtained in BEAST were conducted excluding *R. calligaster* (see Discussion). For the DEC analysis although some ambiguity and possible alternative resolutions exist, the results were consistent with BBM analysis (Appendix S5). Because BMM shows highest resolution at nodes, we considered it most likely for the hypotheses here. Also, the estimation of ancestral area marginal probabilities taking into account phylogenetic uncertainty (Bayesian‐like) has been suggested to reduce uncertainty in the biogeographical reconstruction (Nylander et al., 2008). However, we comment about the major difference of DEC respect to BBM.

**FIGURE 5:**
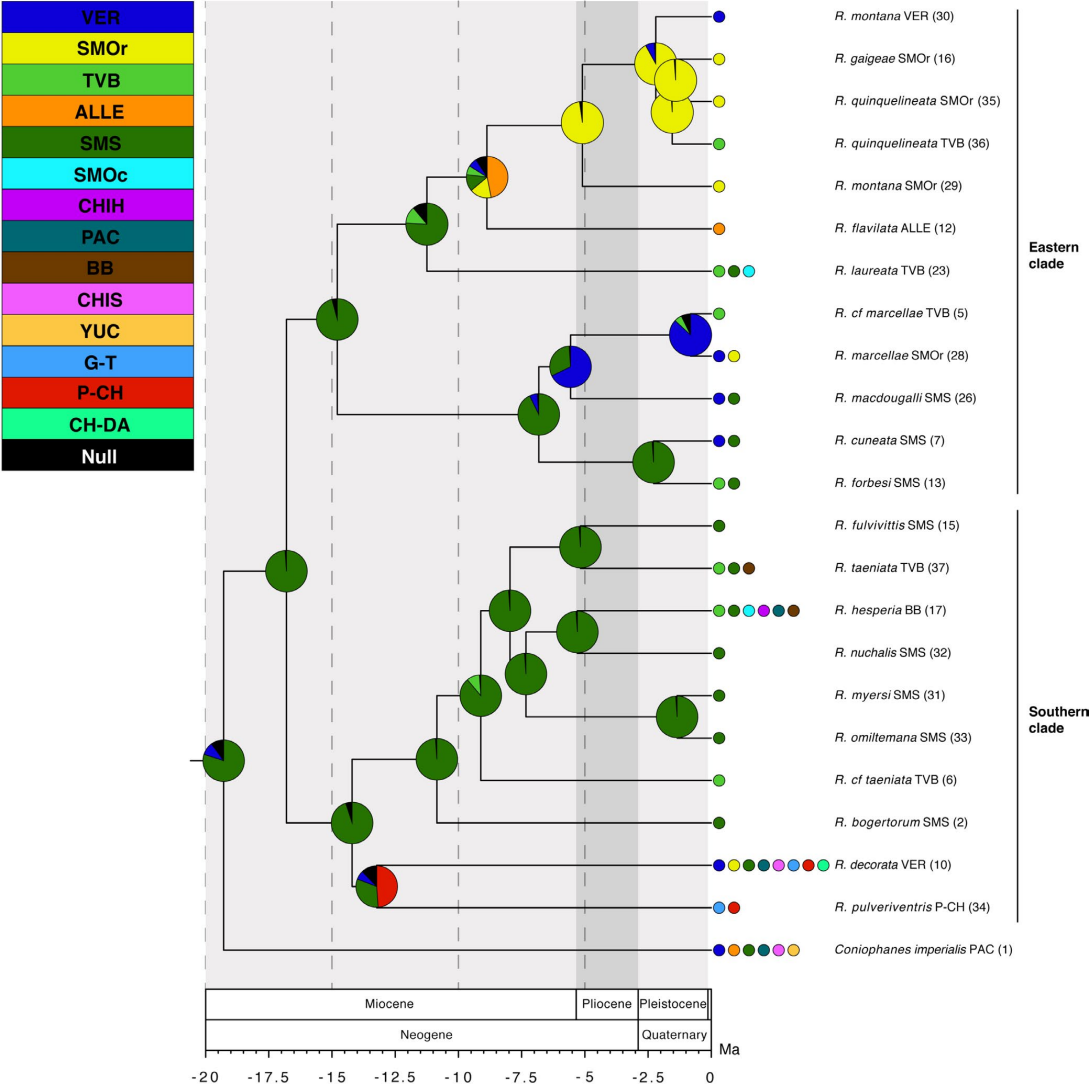
Dated multilocus phylogeny for *Rhadinaea* showing BBM analysis ancestral area reconstructions. Sampled localities are color‐coded to match the biogeographical regions in the inset box. Alleghanian Region (ALLE); Balsas Basin (BB); Chihuahuan Desert (CHIH); Chiapas (CHIS); Chocó‐Darién (CH‐DA); Gatuso‐Talamanca (G‐T); Puntarenas‐Chiriquí (P‐CH); Pacific Lowlands (PAC); Sierra Madre Occidental (SMOc); Sierra Madre Oriental (SMOr); Sierra Madre del Sur (SMS); Trans‐Mexican Volcanic Belt (TVB); Veracruz (VER); Yucatán (YUC); Null, Null range probability. The colored pie charts in the nodes represent the probability of the ancestral area

The RASP analyses showed that the diversification of *Rhadinaea* likely began in the early Miocene in an ancestor widely distributed across the SMS, approximately 16.8 Ma (Figure 5). The Eastern clade distributed across ALLE, SMOc, SMOr, SMS, TVB, and VER split between 0.8 and 14.8 Ma into 12 lineages (Figure 5). During the Miocene, an initial vicariant event in the SMS split the most recent common ancestor (MRCA) of *R. flavilata*, *R. gaigeae* (SMOr), *R. laureata*, *R. montana* (SMOr, VER), and *R. quinquelineata* (SMOr, TVB) from the MRCA of *R. cuneata*, *R. forbesi*, *R. macdougalli*, *R. marcellae,* and *R*. cf. *marcellae* (14.8 Ma), this event was recovered as a dispersal event by DEC; followed by four colonization events during the late Miocene: one by the MRCA of *R. flavilata*, *R. gaigeae* (SMOr), *R*. *montana* (SMOr, VER), and *R. quinquelineata* (SMOr, TVB) from SMS to ALLE (not resolved in DEC), and *R*. *laureata* from SMS to SMOc and TVB (11.2 Ma); followed by the dispersal of the MRCA of *R. gaigeae* (SMOr), *R. montana* (SMOr, VER), and *R. quinquelineata* (SMOr, TVB) from ALLE to SMOr (8.9 Ma); another by the MRCA of *R. macdougalli*, *R. marcellae,* and *R*. cf. *marcellae* from SMS to VER (6.8 Ma); followed by the dispersal of *R. macdougalli* from VER to SMS (5.6 Ma) (Figure 5), for these last two events DEC suggest an ancestral area comparted on SMS and VER.

During the Pliocene, one vicariant event took place in the SMOr between *R. montana* (SMOr) and the MRCA of *R. gaigeae* (SMOr)*, R. montana* (VER), and *R. quinquelineata* (SMOr, TVB) (5.1 Ma). During the Pleistocene, a vicariant event is observed in the SMOr between *R. gaigeae* (SMOr) and *R. quinquelineata* (SMOr) (1.4 Ma), as well as four colonization events (Figure 5): BBM suggests two events from SMOr to VER (*R. montana* VER) (2.2 Ma) and TVB (*R. quinquelineata* TVB) (1.5 Ma); however, DEC suggests an ancestral area of *R. montana* (VER) and *R*. *quinquelineata* (TVB) comparted on SMOr and VER, and SMOr and TVB, respectively; from SMS to TVB and VER, involving *R. forbesi* and *R. cuneata,* respectively (2.3 Ma) (DEC suggests an ancestral area comparted on SMS and VER); and from VER to SMOr and TVB, involving *R. marcellae* and *R*. cf. *marcellae,* respectively (0.8 Ma) (DEC suggests an ancestral area comparted on TVB and VER).

The southern clade distributed across BB, CH‐DA, CHIH, CHIS, G‐T, P‐CH, PAC, SMOr, SMOc, SMS, TVB, and VER split between 1.3 and 14.2 Ma into 10 lineages (Figure 5). During the middle Miocene, a dispersal event is observed from the SMS to P‐CH by BMM and a vicariant even between SMS by DEC, involving the MRCA of *R. decorata* and *R*. *pulveriventris* (14.2 Ma), followed by the dispersal from P‐CH to CH‐DA, CHIS, G‐T, PAC, SMOr, SMS, and VER by *R. decorata* and to G‐T by *R. pulveriventris* (13.2 Ma) (Figure 5). During the late Miocene, a single dispersal event took place from SMS to TVB involving *R. cf taeniata* (9.1 Ma), as well as three vicariant events inside the SMS: a first split between *R. bogertorum* and the MRCA of *R. fulvivittis*, *R. hesperia*, *R. myersi*, *R. nuchalis*, *R. omiltemana*, *R. taeniata,* and *R*. cf. *taeniata* (10.8 Ma); a second split between the MRCA of *R. fulvivittis* and *R. taeniata,* and the MRCA of *R. hesperia*, *R. myersi*, *R. nuchalis,* and *R. omiltemana* (7.9 Ma); and a split between the MRCA of *R. hesperia* and *R. nuchalis* from the MRCA of *R. myersi* and *R. omiltemana* (7.3 Ma) (Figure 5).

During the Pliocene, BBM and DEC recovered two colonization events from SMS to other ancestral areas took place (Figure 5): to BB and TVB (*R. taeniata*) (5.1 Ma); and to BB, CHIH, PAC, SMOc, and TVB (*R*. *hesperia*) (5.3 Ma). During the Pleistocene, a single vicariant event is observed in the SMS between *R. myersi* and *R. omiltemana* (1.3 Ma).

## 4 DISCUSSION

### 4.1 Phylogenetic relationships within *Rhadinaea*

Our molecular‐based phylogeny generally does not support the traditional taxonomy of *Rhadinaea* according to morphology (Myers, 1974, 2011). Our analyses place *Rhadinaea calligaster* as the sister group of *Rhadinella* (Figures 3 and 4). This relationship is not surprising due to the complex morphology within *Rhadinaea* members (Myers, 1974), which makes it difficult to accurately diagnose the members of this genus given the lack of exclusive diagnostic characters and the existence of variable morphological traits, such as the form of the neck collar, pigmentation, and longitudinal patterns of the dorsal scales (Myers, 1974; Sánchez‐García et al., 2019). This situation has resulted in the further modification of the scientific understanding of the composition of the genus (Myers, 2011; Palacios‐Aguilar & García‐Vázquez, 2020). About this, Myers (1974) described the relationships of this monospecific group as obscure and mentioned that morphological features like a bilobated hemipenis, the absence of a subpreocular scale, and a pale bar from the eye to the corner of the mouth indicate an ancestry from the godmani group (now *Rhadinella*). This relationship is plausible, given our results. Therefore, this leads us to infer that *Rhadinaea* is not monophyletic, in contrast with the results obtained by Palacios‐Aguilar and García‐Vázquez (2020). Whereas *Rhadinaea* and *Rhadinella* are recovered as reciprocally monophyletic in their study, here we only retrieved the monophyly of *Rhadinella*, which is yet to be verified including a larger number of taxa.

The classification of the species of *Rhadinaea* into groups defined by Myers (1974, 2011) shows inconsistencies that involve the decorata, flavilata, and taeniata groups. Our phylogenetic hypothesis presented the decorata and taeniata groups as polyphyletic and the flavilata group as paraphyletic (Figures 3 and 4), indicating that the relationships among these species need a further revision due to the low nodal support in the clades containing these species and that they probably do not represent natural groups. Following these observations, we also suggest that the phylogenetic position of *R. calligaster* needs to be revisited in order to confirm the close relationship with *Rhadinella* observed in our analyses, taking into account the evidence that suggests that this group may represent another *Rhadinella* species.

We recovered the majority of the species with more than one sample as monophyletic with strong support values, except for *Rhadinaea marcellae* (Figures 3 and 4). The species that did not show a monophyletic pattern are *R. montana* and *R. quinquelineata*, and appeared to be paraphyletic groups (Figures 3 and 4). This pattern might be the result of an unclear delimitation of these species, as previously suggested by Dixon et al. (2011), with respect to the previously proposed morphological series consisting of *R. gaigeae, R. montana,* and *R. quinquelineata* (Dixon et al., 2011; Myers, 1974), species that show a very similar morphology and are codistributed over the SMOr (Canseco‐Márquez et al., 2004; Medina‐Romero et al., 2016; Myers, 1974). Because of this situation, we decided to include each haplotype of these species in divergence time estimation and ancestral area reconstruction analyses to explore their relationships and diversification. Furthermore, we suggest that additional studies of these taxa including better sampling, additional loci, and explicit testing of alternative species hypotheses using coalescent methods for species delimitation (e.g., Fujita et al., 2012; Yang & Rannala, 2010) are needed to properly address their status and kinship.

Concerning the closest relatives of the genus, it has been shown that *Rhadinaea* is closely related to *Amastridium*, *Coniophanes*, *Rhadinella,* and *Tantalophis* (Daza et al., 2010; Palacios‐Aguilar & García‐Vázquez, 2020; Pyron et al., 2013). Pyron et al. (2013) found a strong relationship between *Rhadinaea* and *Coniophanes* and between both genera and *Rhadinella*, according to Palacios‐Aguilar and García‐Vázquez (2020). In our study, these last two relationships were recovered in the analyses, even though the relationship of *Coniophanes* as the sister group of *Rhadinaea* is not well supported in the BI analysis. We attribute this poorly supported relationship to the differences in our sampling, respecting the number of individuals of each genus. Still, there is more consistent evidence of this kinship in other works that included a larger number of *Coniophanes* samples (Palacios‐Aguilar & García‐Vázquez, 2020). Regarding the relationship with *Rhadinophanes monticola*, in our phylogeny it was recovered as the sister group of *Rhadinella* + *Rhadinaea calligaster* with a low support value in the BI analysis. Due to the complex morphology of this species and uncertainty in its phylogenetic position (Myers & Campbell, 1981), we cannot assess whether this species is more closely related to *Rhadinella* or *Rhadinaea calligaster* given the low support value in our analyses (Figure 3), in which we note that *Rhadinophanes monticola* is not closely related to the main clades of *Rhadinaea* we identified herein (Figures 3 and 4). This outcome could be clarified, considering a broader perspective, including other related colubrid species, as in other studies (e.g., Daza et al., 2010; Pyron et al., 2013).

### 4.2 Historical biogeography

Based on our results of BBM and DEC, it appears that colubrid snakes of the genus *Rhadinaea* have had a relatively long history in North and Central America. The SMS is an extensive mountain system that has been present in the Mexican territory since its formation due to the Laramide geologic activity (DeMets & Stein, 1990) and has presented significant geological changes during the Late Cretaceous to the Miocene (23–100.5 Ma) (Nieto‐Samaniego et al., 2006). This region is known to present a high biological diversity and a high number of endemisms (Blancas‐Calva et al., 2010; Escalante et al., 2002; Navarro‐Sigüenza et al., 2009) including amphibians and reptiles (Flores‐Villela, 1993). About the MRCA of *Rhadinaea* and the first divergences of the main clades herein identified in the SMS during the Miocene (7.3–16.8 Ma) (Figure 5), we infer that these divergences could be due to the discontinuity of the pine–oak forests and cloud forests present in this biogeographical area to date (Rocha‐Méndez et al., 2019; Santiago‐Alvarado et al., 2016), which has experienced changes during similar times (Ornelas et al., 2010, 2013). This mosaic‐like landscape has been associated with centers of diversification along elevational gradients and is believed to be closely related to some divergences between some vertebrate taxa (e.g., *Chlorospingus* [García‐Moreno et al., 2004], *Eupherusa* [Rocha‐Méndez et al., 2019], *Plestiodon* [Pavón‐Vázquez et al., 2018], *Sarcohyla* [Caviedes‐Solis & Leaché, 2018]) within the SMS, probably as a consequence of the climatic changes during the Miocene that produced a long‐term cooling interrupted by warm intervals, especially the middle Miocene climatic optimum (MCO) (17–14 Ma; Zachos, 2001). These divergences predate the diversification of all species inside the Eastern and southern clades and point toward a close relationship between the habitat preferences of the majority of the species of *Rhadinaea* as a primarily montane genus associated with pine–oak and humid forests (García‐Vázquez, Pavón‐Vázquez, et al., 2018; Myers, 1974), and the heterogeneity of the SMS (Bryson et al., 2017; Luna‐Vega et al., 1999; Santiago‐Alvarado et al., 2016).

In this same period, two dispersal events from the SMS to other areas took place. First, we note inside the Eastern clade a northward dispersal of the MRCA of *R. flavilata*, *R. gaigeae*, *R. montana,* and *R. quinquelineata* (Figure 4), which became widespread during the middle Miocene. This event corresponds temporally with the first episode of volcanic and orogenic activity that originated the TVB in the central portion of Mexico (Bryson et al., 2012a, 2012b; Ferrari et al., 2012; Ferrusquía‐Villafranca & González‐Guzmán, 2005; Gómez‐Tuena et al., 2007). This mountain system is known for its influence on the diversification of various montane taxa, creating new montane habitats (Bryson et al., 2012b) and probably allowing further colonization of more mesic‐adapted lineages (e.g., García‐Vázquez, Nieto‐Montes de Oca, et al., 2018; Milstead, 1960) such as *R. flavilata*, in coordination with the low temperatures posterior to the MCO (Zachos, 2001). Secondly, we observe a dispersal event during the Miocene inside the southern clade toward the south by the MRCA of *R. decorata* and *R. pulveriventris* (Figure 5). This divergence is generally consistent with the formation of the Chiapan–Guatemalan highlands of northern Central America, which formed during two different time intervals (Campbell, 1999). The uplift of the extensive northern Central American plateau occurred during the late Miocene to early Pliocene, from approximately 10–3.8 Ma (Rogers et al., 2002). Additionally, the formation in the late Pliocene of a younger chain of volcanoes along the western portion of the Central American plateau (Williams, 1960) had a significant impact on the local biota, both through extinction and the resulting climatic change, creating cloud forest conditions on the windward (south) slopes and rain shadow conditions in the interior valleys (Campbell, 1999). Moreover, an abrupt turnover from xeric, subhumid vegetation to humid forests occurred in south‐central Chiapas and extended along the coast to south‐central Guatemala (Campbell & Vannini, 1988). These wetter conditions over the Pacific coast of southwestern Guatemala and southeastern Chiapas could be suitable for cooler‐adapted lineages such as *Rhadinaea*. Nevertheless, even if this scenario seems plausible, we cannot fully explain the invasion of the MRCA of *R. decorata* and *R. pulveriventris* toward the P‐CH region nested in the lower Central American highlands as a single dispersal event, which is also inhabited by other unsampled species such as *R. sargenti* and *R. vermiculaticeps*. However, there is evidence of a similar colonization pattern in pit vipers (Castoe et al., 2009), where significant biogeographic barriers in Central America, such as the Motagua–Polotchic fault (Marshall, 2007) and the Nicaraguan Depression (Marshall, 2007; Rogers et al., 2002), played an essential role in the diversification of several taxa (Campbell, 1999; Devitt, 2006; Parra‐Olea et al., 2004; Perdices et al., 2005; Savage, 1982), probably this explain the vicariance event found by DEC analysis.

Along with these critical orogenic events, the Miocene climate change appears to have played an important role, sparking evolutionary radiations in some successful modern lineages, including colubrid snakes, and segregating the species along latitudinal and altitudinal environmental gradients (Greene, 1997; Van Devender & Spaulding, 1979). These conditions probably have had an influence in other colonization events during this time within the Eastern and southern clades, such as the colonization of the SMOr by the MRCA of *R. gaigeae, R. montana,* and *R. quinquelineata* (Figure 5), as well as a posterior divergence in the SMOr, perhaps produced by changing ecosystems associated with the wetter climate during this period through the Northern Mexican highlands (Bryson et al., 2013, 2017; Retallack, 2001) as seen in other reptile groups (e.g., *Gerrhonotus* [García‐Vázquez, Nieto‐Montes de Oca, et al., 2018]). Similarly, these changes could influence the dispersal toward the SMOc and the TVB by *R. laureata*, toward VER by the MRCA of *R. macdougalli*, *R. marcellae,* and *R*. cf. *marcellae* and toward SMS by *R. macdougalli* of the Eastern clade; and *R*. cf. *taeniata* of the southern clade (Figure 5). This last hypothetical species is only known from the westernmost portion of the TVB in Sierra San Juan, a too complex area with a high number of endemisms (Escalante & Llorente, 1985; Miranda & Luna‐Vega, 2006).

During the Pliocene, the last episodes of formation of the TVB (3–7.5 Ma) (Ferrari et al., 2012; Gómez‐Tuena et al., 2007) had a role in creating a highland that bisected the southern continuity of the SMOr (Espinosa et al., 2008) and created a complex area of highlands between the Sierra de Juarez, eastern TVB, and southern SMOr. Along with this physiographic change, a series of climatic fluctuations occurred during the Pliocene and Pleistocene (ca. 0.1–4 Ma) (Paillard, 2017; Vanzolini, 1970). These cool intervals are believed to have caused the expansion of some pine, pine–oak, and humid forests to lower elevations due to temperature fluctuations, provoking an extension of the Mexican montane flora to lower elevations of at least 1,000 m (Jaramillo‐Correa et al., 2009; McDonald, 1993; Ornelas et al., 2013; Sosdian & Rosenthal, 2009). Therefore, these orogenic and climatic factors in more recent conjunction are considered to be the processes that have had a more significant impact in a high number of montane taxa (as discussed in Bryson et al., 2012b), and throughout these Pliocene and Pleistocene periods, most extant species of *Rhadinaea* among the Eastern and southern clades originated and colonized other regions.

Within the MTZ, the contact between the SMOr, SMS, TVB, and VER regions is characterized by a very complex biotic interchange, presenting a significant amount of shared floristic and faunistic elements (Espinosa et al., 2004; Marshall & Liebherr, 2000), some of which showed a recent dispersal to adjacent provinces during the Pliocene and Pleistocene (Cavender‐Bares et al., 2011). In this sense, we can attribute the several colonization events in the MTZ involving the eastern species of *R. cuneata, R. forbesi, R. marcellae, R*. cf. *marcellae, R. montana,* and *R. quinquelineata* through some hypothesized filter barriers (Anducho‐Reyes et al., 2008; Bryson, Murphy, et al., 2011; Morafka, 2012), such as in other codistributed taxa (e.g., *Bufo* [Mulcahy et al., 2006], *Gerrhonotus* [García‐Vázquez, Nieto‐Montes de Oca, et al., 2018], *Hyla* [Bryson et al., 2010], *Phrynosoma* [Bryson et al., 2012a], *Sceloporus* [Bryson et al., 2012b] and gymnosperms, angiosperms, and pteridophytes [Luna‐Vega et al., 1999]). For the southern species, this colonization between biogeographical regions in the MTZ is also present in *R. fulvivittis, R. hesperia,* and *R. taeniata* between the BB, CHIH, PAC, SMS, SMOc, and TVB. Meanwhile, these dispersal patterns are observed in other taxa with similar distribution (e.g., *Buarremon* [Navarro‐Sigüenza et al., 2008], *Hyla* [Bryson et al., 2010], *Pituophis* [Bryson, García‐Vázquez, et al., 2011], *Sarcohyla* [Caviedes‐Solis & Leaché, 2018], and *Sceloporus* [Bryson et al., 2012b]) and are believed to be driven by the aforementioned geologic and climatic factors.

Regarding the colonization events by *R. decorata*, we observe a similar southward pattern as in other snake taxa (*Atropoides* and *Cerrophidion* [Castoe et al., 2009]); therefore, we consider this final clade is incomplete due to our sampling with respect to the wide distribution of *R. decorata*. As such, the resolution of the biogeographic patterns also remains incomplete, and this same problem is present within other widely distributed species of *Rhadinaea* such as *R. taeniata* and *R. hesperia*.

Finally, as to the vicariant events involving some of the species present in both Eastern and southern clades, we attribute these divergences to different processes such as soft allopatry through ecological vicariance (Pyron & Burbrink, 2010), given nonidentical lineage ranges of *R. gaigeae* and *R. quinquelineata* in the SMOr, and the action of lowland barriers inside these biogeographical regions, such as river drainages of the Río Grande and the Río Santa Catarina (Pavón‐Vázquez et al., 2018) in the case of the divergence between *R. myersi* and *R. omiltemana* in the SMS. These analyses are enlightening about the geographic origin and timing of most of the *Rhadinaea* species divergences and point toward an origin of the genus more related to the woodland dynamics in the SMS than other proposed biogeographical barriers present in southeastern Mexico such as the IT and ND.

## 5 CONCLUSIONS

Biogeographical studies seek to explain the distribution of species in terms of historical factors and climatic phenomena (García‐Vázquez, Nieto‐Montes de Oca, et al., 2018). The genus *Rhadinaea* has shown to be an insightful model in order to study these factors in a widely distributed group. Extreme climatic oscillations during the Pleistocene, a key driver of diversification between lineages in some taxa (León‐Paniagua et al., 2007), as well as Miocene and Pliocene geomorphology in conjunction with climate change appear to have induced allopatric divergence on a relatively small spatial scale in this genus, and point toward a complex origin inside the heterogeneous area of the SMS and several diversification events among the TVB and adjacent provinces, providing an insight into the historical processes responsible for the diversification in this complex system. The outcome also shows the necessity of further systematic exploration of the genus, as the morphological characters used may not be sufficient to reconstruct the evolutionary history of *Rhadinaea*. On this issue, an integrative perspective using molecular and morphological data, taking into account historical information of the species, might shed some light for the systematics and evolution of these poorly known colubrid snakes.

## CONFLICT OF INTEREST

The authors Uriel Alonso García Sotelo, Uri Omar García Vázquez, and David Nahum Espinosa Organista declare that we have no significant competing financial, professional, or personal interests that might have influenced the performance or presentation of the work described in this manuscript.

## AUTHOR CONTRIBUTIONS

**Uriel A. García‐Sotelo:** Formal analysis (equal); investigation (equal); software (equal); writing‐original draft (equal). **Uri O. García‐Vázquez:** Conceptualization (equal); funding acquisition (equal); investigation (equal); project administration (equal); supervision (equal); validation (equal); writing‐review & editing (equal). **David Espinosa:** Investigation (equal); writing‐review & editing (equal).

## Supporting information

Appendix S1

Appendix S2

Appendix S3

Appendix S4

Appendix S5

## Data Availability

All sequence data generated in this study will be available in the public genetic sequence database GenBank (https://www.ncbi.nlm.nih.gov/genbank/).
