# Supplementary material for: Historical biogeography of the genus Rhadinaea (Squamata: Dipsadinae)
Source: Ecol Evol. 2021 Aug 5;11(18):12413–28. doi: 10.1002/ece3.7988 (PMC8462180; doi:10.1002/ece3.7988)
Supplement: Supplementary file 1 — Appendix S1 [file ECE3-11-12413-s001.docx]

**Appendix S1**. Sampling DNA

Collection and voucher data for *Rhadinaea* genetic samples used in this study. Institutional codes for museum and collections follow Sabaj (2016). AMH, ANMO, CIG, ENS, GK, ISZ, JAC, JRM, MXC, OFV, OORH, RICB, RSB and UOGV are field identifiers for uncatalogued specimens being introduced in the MZFC. The X’s present in the GeneBank accession codes represent pending submissions of sequences, the Em dash represents absent information, asterisks in sample id’s number indicates samples used in divergence time estimation and ancestral area reconstruction analyses. Alleghanian Region (ALLE); Balsas Basin (BB); Chiapas (CHIS); Chihuahuan Plateau (CHIH); Gatuso-Talamanca (G-T); Pacific Lowlands (PAC); Puntarenas-Chiriquí (P-CH); Sierra Madre Occidental (SMOc); Sierra Madre Oriental (SMOr); Sierra Madre del Sur (SMS); Trans-Mexican Volcanic Belt (TVB) and Veracruz (VER).

| **Taxa** | **Sampled area according to this study** | **Locality** | **Voucher Number** | **sequence's Genbank accession number** | | | | **Sample ID** |
| --- | --- | --- | --- | --- | --- | --- | --- | --- |
|  |  |  |  | cmos | cytb | DNAH3 | ND4 |  |
| *Coniophanes imperialis* | PAC | Mexico: Oaxaca: La Cofradía | MZFC-HE 15533 | MZ520160 | MT308778 | MZ520231 | MZ520272 | 1* |
| *Rhadinaea bogertorum* | SMS | Mexico: Oaxaca: Totontepec | CIG 739 | MZ520161 | MZ520199 | MZ520232 | MZ520273 | 2* |
| *Rhadinaea calligaster* | G-T | Costa Rica: Heredia: Volcan Barva | GK 2462 | MZ520162 | MZ520200 | MZ520233 | MZ520274 | 3* |
| *Rhadinaea calligaster* | P-CH | Costa Rica: Cartago: Turrialba | ENS rcalligaster | MZ520163 | MZ520201 | MZ520234 | MZ520275 | 4 |
| *Rhadinaea* cf *marcellae* | TVB | Mexico: Puebla: Xocayucan | ANMO 2109 | MZ520186 | MZ520220 | MZ520257 | MZ520300 | 5* |
| *Rhadinaea* cf *taeniata* | TVB | Mexico: Nayarit: Cerro San Juan | MZFZ 4423 | MZ520164 | MZ520202 | MZ520235 | MZ520276 | 6* |
| *Rhadinaea cuneata* | SMS | Mexico: Veracruz: Aticpac | MZFZ 4424 | MZ520165 | MZ520203 | MZ520236 | MZ520277 | 7* |
| *Rhadinaea decorata* | SMS | Mexico: Oaxaca: Eloxochitlán de Flores Magón | OFV 1109 | MZ520166 | MT308782 | MZ520237 | MZ520278 | 8 |
| *Rhadinaea decorata* | CHIS | Guatemala: Huehuetenango: Barillas | UTA-R 44718 | MZ520167 | MT308780 | MZ520238 | MZ520279 | 9 |
| *Rhadinaea decorata* | VER | Mexico: Veracruz: Otontepec | MZFZ 4425 | MZ520168 | MT308781 | MZ520239 | MZ520280 | 10* |
| *Rhadinaea flavilata* | ALLE | USA: Florida: Laural | CAS-HERP 198634 | AF471152 | AF471078 | — | — | 11 |
| *Rhadinaea flavilata* | ALLE | USA: North Carolina: Southport | MVZ-HERP 164867 | MZ520169 | MZ520204 | MZ520240 | MZ520281 | 12* |
| *Rhadinaea forbesi* | SMS | Mexico: Veracruz: Finca Santa Martha | MZFZ 4426 | MZ520170 | MZ520205 | MZ520241 | MZ520282 | 13* |
| *Rhadinaea forbesi* | TVB | Mexico: Veracruz: Banderillo | JRM 3800 | MZ520171 | MZ520206 | MZ520242 | MZ520283 | 14 |
| *Rhadinaea fulvivittis* | SMS | Mexico: Oaxaca: San Pablo Macuiltianguis | ANMO 2388 | MZ520172 | MZ520207 | MZ520243 | MZ520284 | 15* |
| *Rhadinaea gaigeae* | SMOr | Mexico: San Luis Potosí: Cerro el Cabezón | MZFZ 4427 | MZ520173 | MZ520208 | MZ520244 | MZ520285 | 16* |
| *Rhadinaea hesperia* | BB | Mexico: Guerrero: Olinalá | ANMO 3631 | MZ520174 | MZ520209 | MZ520245 | MZ520286 | 17* |
| *Rhadinaea hesperia* | CHIH | Mexico: Jalisco: La Barca | ISZ 531 | MZ520175 | MZ520210 | MZ520246 | MZ520287 | 18 |
| *Rhadinaea hesperia* | TVB | Mexico: Nayarit: Cerro San Juan | MZFZ 4428 | MZ520176 | MZ520211 | MZ520247 | MZ520288 | 19 |
| *Rhadinaea hesperia* | SMS | Mexico: Guerrero: Malinaltepec | OORH 55 | MZ520177 | MZ520212 | MZ520248 | MZ520289 | 20 |
| *Rhadinaea hesperia* | PAC | Mexico: Nayarit: Bahía de Banderas | RICB 556 | MZ520178 | MZ520213 | MZ520249 | MZ520290 | 21 |
| *Rhadinaea hesperia* | SMS | Mexico: Michoacán: Coalcomán de Vázquez Pallares | AMH 698 | MZ520179 | MZ520214 | MZ520250 | MZ520291 | 22 |
| *Rhadinaea laureata* | TVB | Mexico: Morelos: Huitzilac | MZFC-HE 21661 | MZ520180 | MT308785 | MZ520251 | MZ520292 | 23* |
| *Rhadinaea laureata* | SMS | Mexico: Jalisco: Chilacayote | CIG 503 | MZ520181 | MZ520215 | MZ520252 | MZ520293 | 24 |
| *Rhadinaea laureata* | SMOc | Mexico: Durango: Alemán | CIG 1556 | MZ520182 | MZ520216 | MZ520253 | MZ520294 | 25 |
| *Rhadinaea macdougalli* | SMS | Mexico: Oaxaca: Totontepec | UOGV 2091 | MZ520183 | MZ520217 | MZ520254 | MZ520297 | 26* |
| *Rhadinaea marcellae* | VER | Mexico: Veracruz: Rancho San Nicolasillo | MZFZ 4429 | MZ520184 | MZ520218 | MZ520255 | MZ520298 | 27 |
| *Rhadinaea marcellae* | SMOr | Mexico: San Luis Potosí: Xilitla | ANMO 4339 | MZ520185 | MZ520219 | MZ520256 | MZ520299 | 28* |
| *Rhadinaea montana* | SMOr | Mexico: Tamaulipas: Gómez Farias | CIG 605 | MZ520187 | MZ520221 | MZ520258 | MZ520295 | 29* |
| *Rhadinaea montana* | VER | Mexico: Tamaulipas: Ciudad Victoria | MZFZ 4430 | MZ520188 | MZ520222 | MZ520259 | MZ520296 | 30* |
| *Rhadinaea myersi* | SMS | Mexico: Guerrero: Metlaltónoc | ANMO 3887 | MZ520189 | MZ520223 | MZ520260 | MZ520301 | 31* |
| *Rhadinaea nuchalis* | SMS | Mexico: Guerrero: Atoyac de Álvarez | MZFC-HE 22161 | MZ520190 | MZ520224 | MZ520261 | MZ520302 | 32* |
| *Rhadinaea omiltemana* | SMS | Mexico: Guerrero: Omiltemi | RSB 25 | MZ520191 | MZ520225 | MZ520262 | MZ520303 | 33* |
| *Rhadinaea pulveriventris* | P-CH | Costa Rica: Cártago: Río Grande Orosí | MVZ-HERP 204129 | MZ520192 | MZ520226 | MZ520263 | MZ520304 | 34* |
| *Rhadinaea quinquelineata* | SMOr | Mexico: Puebla: Zacatlán | MZFZ 4431 | — | MZ520227 | MZ520264 | MZ520305 | 35* |
| *Rhadinaea quinquelineata* | TVB | Mexico: Puebla: Hueyapan | MXC-SN QUINQUE | ­— | MZ520228 | MZ520265 | MZ520306 | 36* |
| *Rhadinaea taeniata* | TVB | Mexico: State of Mexico: Valle de Bravo | UOGV 2181 | MZ520193 | MT308787 | MZ520266 | MZ520307 | 37* |
| *Rhadinaea taeniata* | SMS | Mexico: Oaxaca: Santa María Yavesía | MZFC-HE 23859 | MZ520194 | MT308788 | MZ520267 | MZ520308 | 38 |
| *Rhadinella godmani* | CHIS | Guatemala: Quiché: San Juan Cotzal | ENS 7810 | MZ520195 | MZ520229 | MZ520268 | MZ520309 | 39* |
| *Rhadinella hempsteadae* | CHIS | Guatemala: Quiché: Uspantán | UTA-R 42470 | MZ520196 | MT308783 | MZ520269 | MZ520310 | 40* |
| *Rhadinella lachrymans* | CHIS | Guatemala: San Marcos: San Rafael Pie de la Cuesta | UTA-R 42335 | MZ520197 | MT308784 | MZ520270 | MZ520311 | 41* |
| *Rhadinophanes monticola* | SMS | Mexico: Guerrero: Puerto del Gallo | JAC 29554 | MZ520198 | MZ520230 | MZ520271 | MZ520312 | 42* |
| *Achrochordus granulatus* | — | — | NUM-Az 0375 | — | NC_007400 | — | NC_007400 |  |
| *Atheris chlorechis* | — | — | PEM R5297 | AY611921 | AY612012 | — | — |  |
| *Clelia clelia* | — | — | MHNSM 7380 | GQ895803 | GQ895859 | — | — |  |
| *Coluber constrictor* | — | — | CAS-HERP 236039 | MF402211 | MF402798 | MF402397 | — |  |
| *Natrix Natrix* | — | — | MTD T 11570 | — | LL999946 | — | LL999896 |  |

**REFERENCES**

Sabaj, M. H. (2016) Standard symbolic codes for institutional resource collections in herpetology and ichthyology: an online reference. Version 6.5. American Society of Ichthyologists and Herpetologists, Washington, DC. Available from: http://www.asih.org/ (accessed 20 April 2020).
