# Supplementary material for: Historical biogeography of the genus Rhadinaea (Squamata: Dipsadinae)
Source: Ecol Evol. 2021 Aug 5;11(18):12413–28. doi: 10.1002/ece3.7988 (PMC8462180; doi:10.1002/ece3.7988)
Supplement: Supplementary file 2 — Appendix S2 [file ECE3-11-12413-s003.docx]

**Appendix S2**. Technical Details on DNA Sequencing and Sequence Edition

**DNA extraction, PCR, sequencing and sequence edit.** Total genomic DNA was extracted from liver, muscle or shed skins following the main protocol of ammonium acetate by Fetzner (1999) or using the DNeasy Blood & Tissue Kit (QIAGEN). All gene regions were amplified via polymerase chain reaction (PCR) in a 25 µl reaction volume containing 0.5–1.0 µl deoxynucleoside triphosphates (dNTPs; 2-10 mM) (ThermoFisher Scientific, NV, USA), 18–19.25 µl double-distilled water, 0.2–0.5 µl each primer (2-10 mM), 2.5 µl 1 X PCR buffer, 1.2 mM MgCl2 (ThermoFisher Scientific), 0.15 µl Taq DNA polymerase (ThermoFisher Scientific), and 1.0–1.5 µl template DNA. For cmos and DNAH3, DNA was denatured at 94 °C for 3 min, followed by 35 cycles of 94 °C for 45 s, 48–55 °C for 45 s, and 72 °C for 1 min. A final extension phase of 72 °C for 6 min terminated the protocol (Saint et al., 1998). For cytb, DNA was denatured at 94 °C for 7 min, followed by 40 cycles of 94 °C for 30 s, 46 °C for 30 s, and 72 °C for 1 min. A final extension phase of 72 °C for 7 min terminated the protocol (Burbrink et al., 2000). For ND4, DNA was denatured at 94 °C for 3 min, followed by 35 cycles of 93 °C for 30 s, 50 °C for 1 min, and 72 °C for 1 min. A final extension phase of 72 °C for 7 min terminated the protocol (Forstner et al., 1995). The primers used for amplification are presented in the following table.

Used primers in the present study to amplify cmos, cytb, DNAH3 and ND4 genes for Rhadinaea. *Primers used for sequencing.

| Gene | Primer name | Primer sequence | Reference |
| --- | --- | --- | --- |
| Cyt-b | L14919* | 5'-AAC CAC CGT TGT TAT TCA ACT-3' | Burbrink et al., 2000 |
|  | L15584-Z* | 5'-CCA TTY CAC CCM TAY CAC TC-3' | De Queiroz et al., 2002 |
|  | H15716-Z | 5'-GGT TTY ATG TGR TGT GGT GTT AC-3' | Slowinski & Lawson, 2002 |
|  | H16064* | 5'-CCT TGG TTT ACA AGA ACA ATG CTT TA-3' | Burbrink et al., 2000 |
| ND4 & tRNA’s | ND4* | 5’-TGA CTA CCA AAA GCTC ATG TAG AAG C-3’ | Forstner et al., 1995 |
|  | Leu* | 5’TRC TTT TAC TTG GAT TTG CAC CA-3’ | Forstner et al., 1995 |
|  | GerrIntFwd* | 5’-AAY ACT AAC TAY GAA CGA AC-3’ | The present study |
|  | GerrIntRev | 5’-TGT GTT GGR AGT TTT CCT CGT TG-3’ | The present study |
|  | RhaIntRev* | 5’- CCT GTG AAG TTT ATR CTK GG-3’ | The present study |
| DNAH3 | DNAH3-f1 | 5'-GGT AAA ATG ATA GAA GAY TAC TG-3' | Townsend et al., 2008 |
|  | DNAH3-r6* | 5'-CTK GAG TTR GAH ACA ATK ATG CCA T-3' | Townsend et al., 2008 |
| C-mos | S77* | 5'-CAT GGA CTG GGA TCA GTT ATG-3' | Lawson et al., 2005 |
|  | G74 | 5'-TGA GCA TCC AAA GTC TCC AAT C-3' | Saint et al. 1998 |

Double-stranded PCR amplified products were checked by electrophoresis on a 1% agarose gel. PCR products were purified with polyethylene glycol precipitation (Lis, 1980). DNA templates were sequenced in both directions with the Big Dye Terminator v. 3.1 cycle sequencing kit (Applied Biosystems, Inc.), as well as an ABI 3100 automated DNA sequencer (Applied Biosystems, Inc.) by Macrogen Korea Inc., using the amplification primers (see table above). Sequences were assembled and edited in the software Sequencher 3.1.1 (Nishimura, 2000), edited sequences were aligned per gene in MEGA7 (Kumar et al., 2016), using Muscle algorithm (Edgar, 2004), and a concatenated matrix was created later using Mesquite 3.61 (Maddison & Maddison, 2019).

**REFERENCES**

Burbrink, F. T., Lawson, R., & Slowinski, J. B. (2000). Mitochondrial DNA phylogeography of the polytypic North American rat snake (*Elaphe obsoleta*): a critique of the subspecies concept. *Evolution*, 54(6), 2107-2118. <https://doi.org/10.1111/j.0014-3820.2000.tb01253.x>

de Queiroz, A., Lawson, R., & Lemos-Espinal, J. A. (2002). Phylogenetic relationships of North American garter snakes (Thamnophis) based on four mitochondrial genes: how much DNA sequence is enough? *Molecular phylogenetics and evolution*, 22(2), 315-329. <https://doi.org/10.1006/mpev.2001.1074>

Edgar, R. C. (2004). MUSCLE: multiple sequence alignment with high accuracy and high throughput. *Nucleic acids research*, 32(5), 1792-1797. https://doi.org/10.1093/nar/gkh340

Fetzner Jr, J. W. (1999). Extracting high-quality DNA from shed reptile skins: a simplified method. *Biotechniques*, 26(6), 1052-1054.

Forstner, M. R., Davis, S. K., & Arévalo, E. (1995). Support for the hypothesis of anguimorph ancestry for the suborder Serpentes from phylogenetic analysis of mitochondrial DNA sequences. *Molecular Phylogenetics and Evolution*, 4(1), 93-102. <https://doi.org/10.1006/mpev.1995.1010>

Kumar, S., Stecher, G., & Tamura, K. (2016). MEGA7: molecular evolutionary genetics analysis version 7.0 for bigger datasets. *Molecular biology and evolution*, 33(7), 1870-1874. https://doi.org/10.1093/molbev/msw054

Lawson, R., Slowinski, J. B., Crother, B. I., & Burbrink, F. T. (2005). Phylogeny of the Colubroidea (Serpentes): new evidence from mitochondrial and nuclear genes. *Molecular phylogenetics and evolution*, 37(2), 581-601. https://doi.org/10.1016/j.ympev.2005.07.016

Lis, J. T. (1980) Fractionation of DNA fragments by polyethylene glycol induced precipitation. *Methods in Enzymology*, 65(1), 347-353.

Maddison, W. P., & Maddison, D. R. (2019). Mesquite: a modular system for evolutionary analysis. Version 3.61 http://www.mesquiteproject.org

Nishimura, D. (2000). Sequencher 3.1. 1. *Biotech Software & Internet Report*, *1*, 24-30.

Saint, K. M., Austin, C. C., Donnellan, S. C., & Hutchinson, M. N. (1998). C-mos, a nuclear marker useful for squamate phylogenetic analysis. *Molecular phylogenetics and evolution*, 10(2), 259-263.

Slowinski, J. B., & Lawson, R. (2002). Snake phylogeny: evidence from nuclear and mitochondrial genes. *Molecular Phylogenetics and Evolution*, 24(2), 194-202. https://doi.org/10.1016/S1055-7903(02)00239-7

Townsend, T. M., Alegre, R. E., Kelley, S. T., Wiens, J. J., & Reeder, T. W. (2008). Rapid development of multiple nuclear loci for phylogenetic analysis using genomic resources: an example from squamate reptiles. *Molecular phylogenetics and evolution*, 47(1), 129-142. https://doi.org/10.1016/j.ympev.2008.01.008
