# Supplementary material for: Historical biogeography of the genus Rhadinaea (Squamata: Dipsadinae)
Source: Ecol Evol. 2021 Aug 5;11(18):12413–28. doi: 10.1002/ece3.7988 (PMC8462180; doi:10.1002/ece3.7988)
Supplement: Supplementary file 3 — Appendix S3 [file ECE3-11-12413-s005.docx]

**Appendix S3**. Technical Details on Fossils Utilized for the Divergence Time Estimates

**Fossils Included for the Divergence Time Estimates**

(1) *Procerophis sahnii* (Rage et al., 2008) 50.5-93.9 Ma (Ypresian to Cenomanian), to calibrate the most recent common ancestor (MRCA) of Pan-Colubroidea (*Achrochordus* + Xenodermatidae) as discussed in Head et al. (2016), which was defined under a lognormal distribution with an offset value of 50.5, a mean of 2.48 and a standard deviation of 1.0, resulting in a distribution with a mean of 62.4 Ma and a 95% prior credible interval from the Ypresian at 52.8 Ma to Albian at 112 Ma.

(2) Colubrid Indet. (Smith, 2013) 35.2-54 Ma (Rupelian to Ypresian), to calibrate the MRCA of Colubroidea as discussed in Zaher et al. (2019), which was defined under a lognormal distribution with an offset value of 33.9, a mean of 2.8 and a standard deviation of 0.8, resulting in a distribution with a mean of 50.3 Ma and a 95% prior credible interval from the Priabonian at 34.7 Ma to the Cenomanian at 95.2 Ma.

(3) *Paleoheterodon tiheni* (Holman, 1977) 12.5-54 Ma (Serravallian to Ypresian), to calibrate the MRCA of Dipsadidae as discussed in Zaher et al. (2019), which was defined under a lognormal distribution with an offset value of 12.5, a mean of 2.2 and a standard deviation of 1.0, resulting in a distribution with a mean of 21.5 Ma and a 95% prior credible interval from Langhian at 14.2 Ma to Selandian at 59.3 Ma.

(4) Dipsadinid Indet. (Camolez and Zaher, 2010; Onary et al., 2017) 0.129-2.58 Ma (Chibanian to Gelasian), to calibrate the MRCA of Dipsadinae, which was defined under a lognormal distribution with an offset value of 0.129, a mean of 1.5 and a standard deviation of 1.0, which resulted in a distribution with a mean of 4.61 Ma and a 95% prior credible interval from Calabrian at 0.99 Ma to Chattian at 23.3 Ma. This last fossil specimen was found in a sedimentary deposit in central Brazil (Nossa Senhora Aparecida e Carneiro cavern) dated from upper Pleistocene (Camolez and Zaher, 2010). The specimen consists of two articulated trunk vertebrae, showing a distinctive, laterally expanded interzygapophyseal margins (or *margo lateralis*). The authors discuss that this typically expanded *margo lateralis* is present only in another Dipsadinae snakes, such as *Imantodes, Atractus, Dipsas, Leptodeira* and *Sibynomorphus*. Due to the lack of other vertebral or cranial material the authors explain that this specimen cannot be assigned to any genera. Anyways, the Dipsadinae vertebral features are easily identificated (Camolez and Zaher, 2010).

**REFERENCES**

Camolez, T., & Zaher, H. (2010). Levantamento, identificação e descrição da fauna de Squamata do Quaternário brasileiro (Lepidosauria). *Arquivos de Zoologia*, 41(1), 1-96. https://doi.org/10.11606/issn.2176-7793.v41i1p1-96

Head, J. J. (2015). Fossil calibration dates for molecular phylogenetic analysis of snakes 1: Serpentes, Alethinophidia, Boidae, Pythonidae. *Palaeontologia Electronica*, 18(1), 1-17.

Holman, J. A. (1977). Upper Miocene snakes (Reptilia, Serpentes) from southeastern Nebraska. *Journal of Herpetology*, 11(3), 323-335. https://doi.org/10.2307/1563245

Onary, S. Y., Fachini, T. S., & Hsiou, A. S. (2017). The Snake Fossil Record from Brazil. *Journal of Herpetology*, 51(3), 365-374. https://doi.org/10.1670/16-031

Rage, J. C., Folie, A., Rana, R. S., Singh, H., Rose, K. D., & Smith, T. (2008). A diverse snake fauna from the early Eocene of Vastan Lignite Mine, Gujarat, India. *Acta Palaeontologica Polonica*, 53(3), 391-403. <https://doi.org/10.4202/app.2008.0303>

Smith, K. T. (2013). New constraints on the evolution of the snake clades Ungaliophiinae, Loxocemidae and Colubridae (Serpentes), with comments on the fossil history of erycine boids in North America. *Zoologischer Anzeiger-A Journal of Comparative Zoology*, 252(2), 157-182. <https://doi.org/10.1016/j.jcz.2012.05.006>

Zaher, H., Murphy, R. W., Arredondo, J. C., Graboski, R., Machado-Filho, P. R., Mahlow, K., ... Zhang, Y. P. (2019). Large-scale molecular phylogeny, morphology, divergence-time estimation, and the fossil record of advanced caenophidian snakes (Squamata: Serpentes). *PloS one*, 14(5), e0216148. https://doi.org/10.1371/journal.pone.0216148
