# Supplementary material for: Historical biogeography of the genus Rhadinaea (Squamata: Dipsadinae)
Source: Ecol Evol. 2021 Aug 5;11(18):12413–28. doi: 10.1002/ece3.7988 (PMC8462180; doi:10.1002/ece3.7988)

**Appendix S4.** Genealogies obtained from each individual data matrix using ML (odd numbers) and IB (even numbers) for C-mos (1, 2), Cytb (3, 4), DNAH3 (5, 6) and ND4 (7, 8). Black dots represent poorly supported nodes (bootstrap value ≥70; Bayesian posterior probability value ≥0.95). Numbers at dots are bootstrap/Bayesian posterior probability values for these nodes.


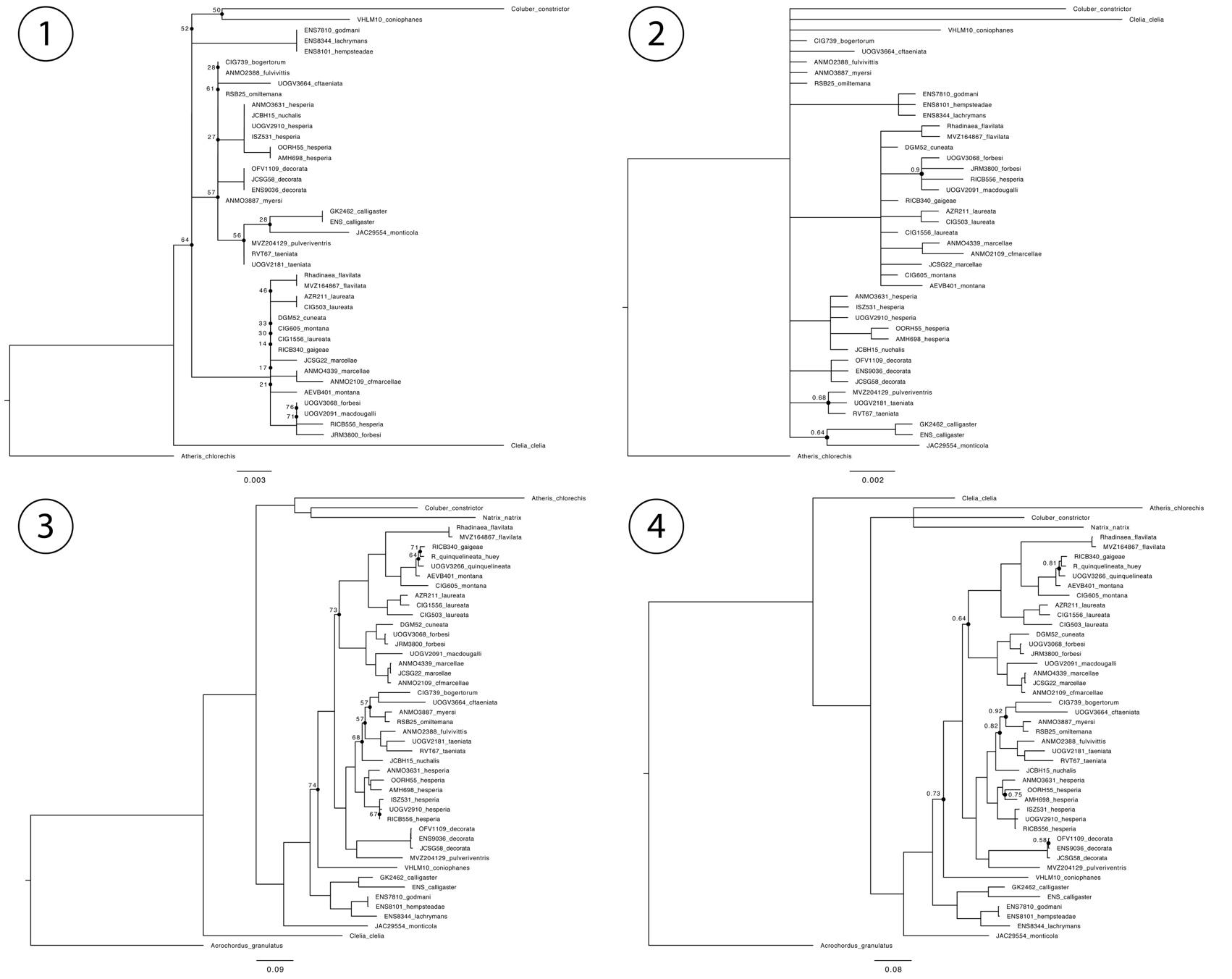


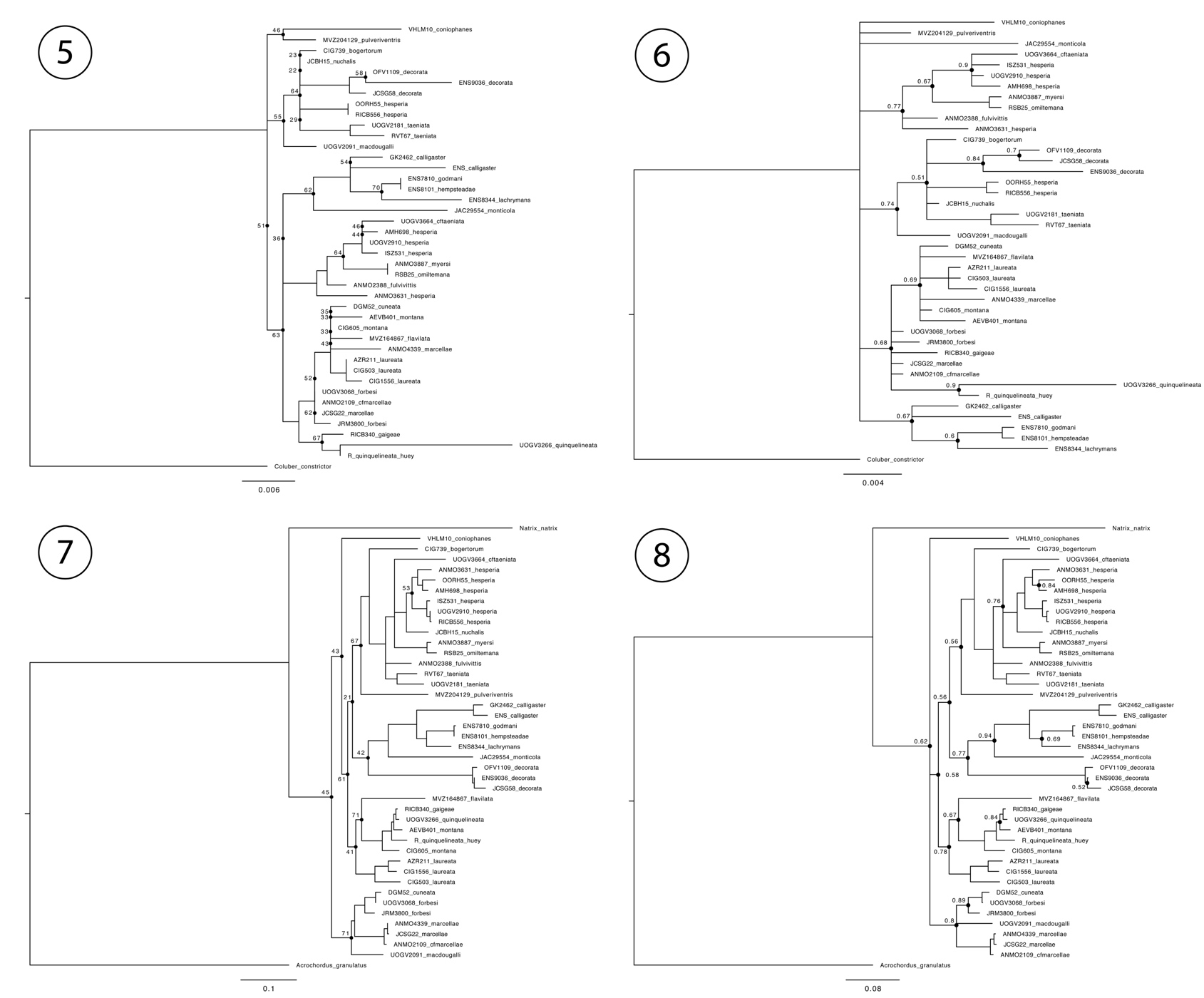

Supplement: Supplementary file 4 — Appendix S4 [file ECE3-11-12413-s004.docx]
