# Supplementary material for: Historical biogeography of the genus Rhadinaea (Squamata: Dipsadinae)
Source: Ecol Evol. 2021 Aug 5;11(18):12413–28. doi: 10.1002/ece3.7988 (PMC8462180; doi:10.1002/ece3.7988)
Supplement: Supplementary file 5 — Appendix S5 [file ECE3-11-12413-s002.docx]

**Appendix S5**

Ancestral area reconstruction of *Rhadinaea* main clades using the dispersal-extinction-cladogenesis (DEC) model (Ree et al., 2005; Ree and Smith, 2008) implemented in RASP (Smith et al., 2010). Alternative ancestral ranges of nodes (with frequency occurrence) are shown in pie chart form. Colors of the charts correspond to the most likely ancestral areas inferred. Letters represent the following biogeographic regions: (A) Alleghanian, (B) Sierra Madre del Sur, (C) Gatuso-Talamanca, (D), Transmexican Volcanic Belt, (E) Chiapas, (F) Veracruz, (G) Sierra Madre Oriental, (H) Balsas Basin, (I) Chihuahuan plateau, (J) Pacific Lowlands, (K) Sierra Madre Occidental, (L) Puntarenas-Chiriquí, (M) Chocó-Darién, (N) Yucatán).


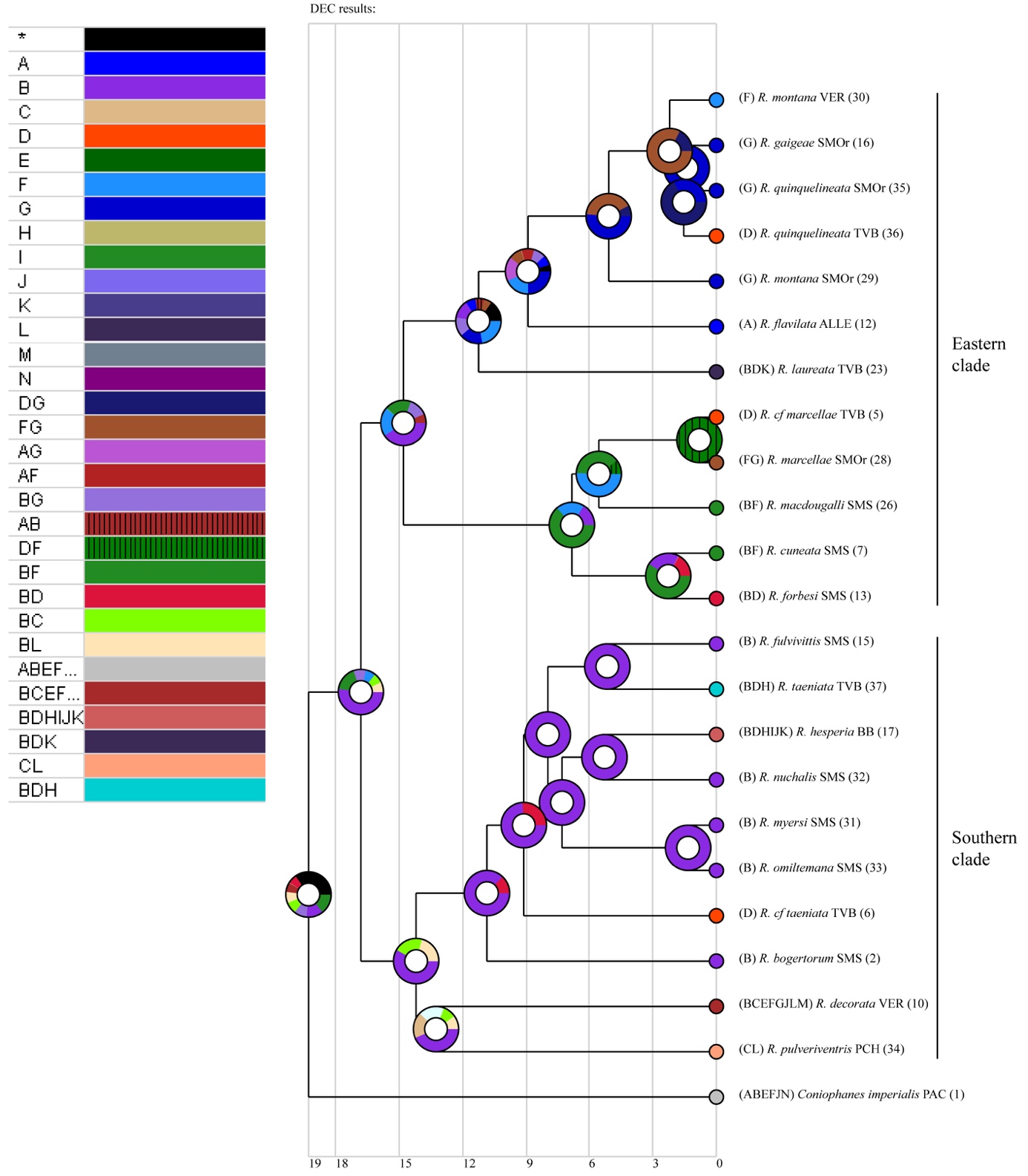


Ree, R. H., Moore, B. R., Webb, C. O., & Donoghue, M. J. (2005). A likelihood framework for inferring the evolution of geographic range on phylogenetic trees. Evolution, 59(11), 2299-2311. xhttps://doi.org/10.1111/j.0014-3820.2005.tb00940.x

Ree, R. H., & Smith, S. A. (2008). Maximum likelihood inference of geographic range evolution by dispersal, local extinction, and cladogenesis. Systematic biology, 57(1), 4-14. https://doi.org/10.1080/10635150701883881
